# RosettaAMRLD: A Reaction-Driven Approach for Structure-Based Drug Design from Combinatorial Libraries with Monte Carlo Metropolis Algorithms

**DOI:** 10.1021/acs.jcim.5c00497

**Published:** 2025-06-11

**Authors:** Yidan Tang, Rocco Moretti, Jens Meiler

**Affiliations:** † Department of Chemistry, 5718 Vanderbilt University, Nashville, Tennessee 37240, United States; ‡ Center for Structural Biology, Vanderbilt University, Nashville, Tennessee 37240, United States; § Institute for Drug Discovery, Faculty of Mathematics and Informatics, Faculty of Chemistry and Mineralogy, University of Leipzig, Leipzig 04103, Germany; ∥ Center for Scalable Data Analytics and Artificial Intelligence ScaDS.AI and School of Embedded Composite Artificial Intelligence SECAI, Dresden/Leipzig 04103, Germany; ⊥ Department of Pharmacology, Institute of Chemical Biology, Center for Applied Artificial Intelligence in Protein Dynamics, Vanderbilt University, Nashville, Tennessee 37240, United States

## Abstract

The Rosetta automated Monte Carlo reaction-based ligand design (RosettaAMRLD) integrates a Monte Carlo Metropolis (MCM) algorithm and reaction-driven molecule proposal to enhance structure-based *de novo* drug discovery. By leveraging combinatorial ultralarge libraries, RosettaAMRLD ensures synthetic accessibility, optimizing protein–ligand interactions while efficiently sampling accessible chemical space. Importantly, RosettaAMRLD can be initiated without a known binder, broadening its applicability to novel pharmaceutical targets. We applied RosettaAMRLD to three protein classes typically targeted by drugs, demonstrating its ability to generate novel, synthetically accessible ligands with active-like binding poses. Benchmark results show that RosettaAMRLD can propose diverse ligands with significantly improved docking scores compared to random sampling, and multiround iteration further enhances output quality, resulting in molecules with *in silico* properties exceeding those of known actives. The method’s capability to explore ultralarge chemical spaces and generate novel drug-like molecules highlights its potential in early stage drug discovery.

## Graphical abstract

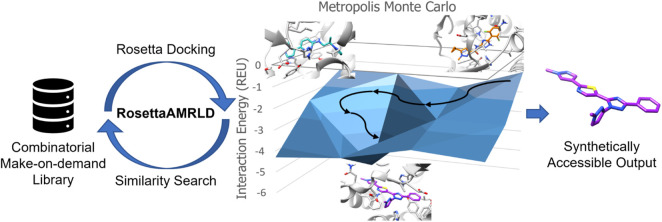

## 1 Introduction

Drug discovery is a resource-intensive process, often taking a better part of a decade and costing between $161 million and $4.54 billion to bring a new drug to market.^1,2^ Nearly half of these costs are incurred during the early stages, which traditionally rely on high-throughput screening (HTS) to identify potential drug candidates.^3,4^ Over the past two decades, computer-aided drug design (CADD) has emerged as a compelling alternative, significantly reducing screening costs while increasing hit rates and expanding the diversity of compounds searched.^5^ CADD methods are broadly categorized into two types: structure-based (SB) and ligand-based (LB). SB approaches simulate interactions between small molecules and the target protein structure, whereas LB approaches infer structure–activity relationships from known binders and nonbinders.^5^ SB methods rely on the availability of structural information and are particularly powerful when high-resolution protein structures are available, as they provide target-specific insights that are not accessible through LB methods.

The field of SB-CADD has seen significant advancements as more and more X-ray, nuclear magnetic resonance, and electron microscopy structures of therapeutic targets become available. The development of homology modeling has further expanded the utility of SB methods by enabling predictions of protein structures based on sequence similarity.^6,7^ In recent years, the number of target structures available for use in SB methods has dramatically increased due to the success of AlphaFold’s deep-learning models in predicting protein structures.^8,9^ This proliferation of structural data presents unprecedented opportunities for SB approaches in drug discovery.

Structure-based virtual screening (SBVS) has been widely applied to numerous proteins and has contributed to the discovery of several approved drugs.^10^ However, despite advancements in computational hardware, the evaluation of protein–ligand interactions remains computationally expensive, limiting the practical size of chemical libraries that can be screened. The drug-like chemical space is estimated to be 10^60^ or more, whereas current SBVS campaigns can only screen libraries with up to 10^9^ molecules.^11,12^ Given the scale of this search problem, exhaustive exploration is impractical, and the need arises for more efficient strategies to navigate the vast space.

SB *de novo* drug design offers an attractive solution by generating new molecules with pharmacological potential from scratch, based on the target protein structure.^13^ Unlike SBVS, which is limited to existing chemical libraries, *de novo* design can explore a much broader chemical space and provide novel starting points for further optimization. SB *de novo* design methods have advanced significantly in recent years,^14^ including the use of Monte Carlo Metropolis (MCM) algorithms.^15,16^ Early MCM methods in the field constructed ligands by incrementally adding or removing individual atoms.^15^ As the field shifted from atom-based to fragment-based methods in the early 2000s,^17^ newer MCM implementations assemble molecules in a similar fashion, but through the addition or removal of predefined fragments.^16^ However, balancing chemical novelty with synthetic accessibility still remains a significant challenge across the SB *de novo* design landscape. Existing methods are often confined to the chemical space of known active molecules or to proposals of multistep synthetic routes to a final product, where low yield and poor synthetic feasibility hinder further experimental validation. While some approaches incorporate synthetic accessibility filters,^18^ they often fail to fully integrate this consideration into the design process.^14^

Recent advancement in efficient exploration of ultralarge, synthetically accessible chemical spaces have increasingly leveraged reaction-based algorithms. Klarich et al. introduced an active learning approach using Thompson Sampling to iteratively sample reagents and update their probabilistic distributions. Their method can be integrated with either SB or LB evaluation strategies, enabling the identification of promising candidates without exhaustive enumeration of the entire combinatorial library.^19^ Swanson et al. developed a LB approach, using Monte Carlo Tree Search guided by a machine learning model trained on experimental activity data to construct and evaluate antibiotic candidates from a combinatorial space.^20^ The reaction-driven design ensures that the generated molecules can be synthesized and tested *in vitro*, leading to 6 out of 58 tested molecules with validated antibacterial activities. These studies underscore the growing utility of reaction-based frameworks in drug discovery and set a strong precedent for further innovation.

In this study, we present Rosetta Automated Monte Carlo Reaction-based Ligand Design (RosettaAMRLD), a SB *de novo* design method. RosettaAMRLD integrates the MCM algorithm with a single-step reaction-driven approach to generate synthetically accessible compounds. More broadly, RosettaLigand has been a very successful example of applying MCM to flexible ligand docking.^21,22^ Building on these past efforts, this method combines the power of iterative sampling with the flexible ligand docking capabilities of the Rosetta software suite to generate drug-like molecules efficiently. By avoiding complex multistep synthesis—a hallmark of traditional approaches—RosettaAMRLD ensures that each newly proposed molecule is chemically feasible by drawing directly from combinatorial libraries of readily accessible fragments. Unlike existing MCM methods that generate molecules through conventional fragment-based modifications, RosettaAMRLD adopts a similarity-guided strategy to prioritize chemical motifs aligned with target-binding preferences. This novel approach allows more flexibility in sampling the chemical space while also having the capacity to explore ultralarge chemical libraries. Recently advances in combinatorial make-on-demand libraries have leveraged both synthetic accessibility and chemical diversity, offering immense opportunities for SB methods. The Enamine REAL space, for instance, has surpassed 60 billion compounds, and this number continues to grow.^23^ By leveraging these combinatorial ultralarge libraries, RosettaAMRLD can explore a vast chemical space while maintaining a focus on synthetic accessibility. Here, we demonstrate RosettaAMRLD’s versatility in generating novel, synthetically accessible ligands across three protein targets: tRNA (guanine-N(1))-methyltransferase (TrmD), cyclin-dependent kinase 2 (CDK2), and Orexin receptor type 1 (OX1R). At the time of benchmarking in this study, we used an earlier version of the Enamine REAL space comprising over 30 billion compounds. The benchmark results show that RosettaAMRLD outperforms random sampling, consistently delivering molecules with superior docking scores. Moreover, we observe that the top-scoring designs retain key protein–ligand interactions found in known actives, underscoring its effectiveness in SB drug design.

## 2 Results

### 2.1 Overview of RosettaAMRLD

The protocol requires two primary inputs: a protein–ligand complex and a combinatorial library with predefined reactions and corresponding reagents. The protein–ligand complex must include a defined binding pocket and can be prepared from experimental structures or docking results. The initial complex can be a poorly docked one or with a low-affinity ligand, so long as it provides a starting point for the exploration in the chemical space. The MCM algorithm adopted here is an iterative process (Figure 1). Each iteration of the protocol consists of three key steps: (1) ligand generation through similarity-guided fragment sampling; (2) alignment to the previous ligand; and (3) evaluation of ligand binding. All proposed ligands at every iteration are reaction products directly from the combinatorial library, therefore ensuring synthetic accessibility throughout the process. The sampling process simulates a Markov Chain-like exploration of the chemical space, where successive ligand candidates are guided by compound similarity and energy optimization. The protocol incorporates a dynamic sampling strategy that adaptively balances local refinement with broader exploration, improving convergence and sampling efficiency. The RosettaAMRLD protocol can be repeated multiple times with a cascaded sampling workflow, extending only the best-performing routes to efficiently explore the chemical space and escape local minima, thereby enhancing the effectiveness of the optimization process. The protocol is implemented within the Rosetta software suite and can be fully customized using RosettaScripts, an XML-based language,^24^ which allows users to tailor the design process to specific design objectives.

**Figure 1:**
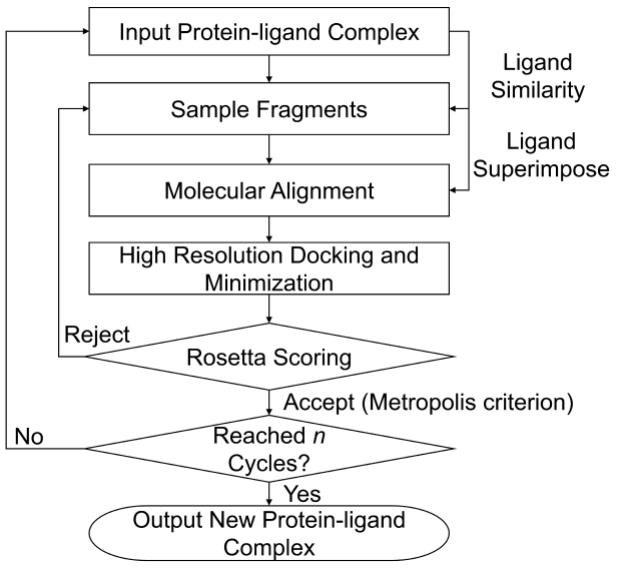
Flowchart of RosettaAMRLD.

### 2.2 Reaction-Based Ligand Generation Guided by Similarity

RosettaAMRLD constructs new ligand candidates by sampling fragments from a combinatorial library based on their structural similarity to a reference or input molecule. The process begins by encoding both the input molecule and the fragments in the library using RDKit Daylight-like Fingerprints.^25,26^ The fragments in the library are then ranked based on their similarity to the reference molecule. Since the fragments are typically smaller in size than the reference molecule, we use the Tversky index, an asymmetric index to calculate the similarities^27^ (see Supporting Information Section 2). We then employ a geometrically weighted sampler (see below) to iteratively move fragments into corresponding “reaction containers”, with top-ranking ones more likely to be chosen. Each “reaction container” corresponds to one of the predefined reactions and holds a certain number of “sub-containers” that corresponds to the number of components in that reaction. Fragments that can undergo the reaction are collected by the corresponding component “sub-containers”. A potential product can be generated by virtually running the reaction with one fragment from each of the component “sub-containers”. All possible products from these “reaction containers” are collected as “candidate molecules”. The fragment sampling step continues until the number of candidate molecules reaches a user-set limit. The candidate sampling step is similar to the fragment sampling step, but the Tanimoto similarity is employed instead.^28^ A similarity score to the reference molecule is calculated for each candidate and the final ligand candidate is selected with geometrically weighted sampling. The geometrically weighted sampling approach ensures that more similar candidates are favored, but less similar ones are still considered, promoting diversity in the outputs. A schematic of the ligand generation step is shown in Figure 2. Our sampling strategy accommodates an arbitrary number of distinct reactions, each with an arbitrary number of reacting components, including reactions with three or more components. Each reaction component is drawn from an independent list of arbitrary length.

**Figure 2:**
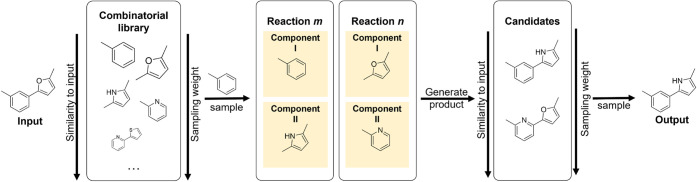
Schematic of the reaction-based ligand generation step.

### 2.3 Geometrically Weighted Sampling Adaptive to Varying Sizes of Chemical Libraries

We strategically avoid a deterministic design route and aim to cover a diverse set of molecules by starting multiple routes from the same state. Nevertheless, the design route should still be guided by structural similarity. Therefore, we employ a sampler to draw with higher frequencies fragments or molecules that are more similar to the reference molecule. A weighted sampler using similarity scores directly as weights does not work well with a skewed distribution where most molecules have a low similarity score. A desired sampler should assign the majority of the weight to the most similar molecules, even with the presence of a large number of low similarity molecules. In this sense, the similarity score ranking instead of the scores themselves gives a more robust basis for assigning weights, since ranking disregards the original skewed score distribution. To ensure that higher weights are assigned to higher ranks, we apply a geometric distribution where the weights decay down the rank. The rate of decay is decided by the total number of molecules and an adjustable parameter which controls the weight proportion that is assigned to the top-ranking molecules. This novel sampler ensures that the majority of the weight is assigned to the most similar molecules, while still maintaining a nonzero probability of selecting lower-ranked ones. The adaptive rate of decay secures a robust sampling performance across chemical libraries of various sizes.

With a list of molecules sorted based on similarity to the reference molecule and a total sampling weight of 1, we define a sampling ratio 0 < *x* < 1 as the fraction of the list where the top *x* proportion is assigned a cumulative sampling weight of 1 – *x* (for instance, a sampling ratio of 0.1 means the top 10% molecules by similarity account for 90% of the weight during sampling; a sampling ratio of 0.01 means the top 1% molecules account for 99% of the weight). The weights follow a geometric distribution and the *k*-th molecule has a weight of

$$a_{k}=\left(1-\sqrt[{xN}]{x}\right){\left(\sqrt[{xN}]{x}\right)}^{k-1}$$

where *N* is the total number of molecules in the list. (See Supporting Information Section 1 for a detailed mathematical derivation.)

The adjustable sampling ratio controls how the sampling weights are distributed between the molecules, directly affecting the likelihood of selecting less similar molecules. To verify this, we simulated the sampling process independently of the MCM framework. In this simulation, we compared three samplers—a geometrically weighted sampler based on ranks of similarity scores, a weighted sampler using similarity scores directly as weights, and a random sampler—by drawing 1,000 independent samples with each sampler from the Enamine REAL space. We set the sampling ratio of the geometrically weighted sampler to 0.1, 0.01, and 0.001 to evaluate the impact on the sampling performance. The results showed that the geometrically weighted sampler successfully biased the selection toward highly similar molecules, performing significantly better than the weighted and random samplers. More importantly, the degree of bias can be adjusted by varying the sampling ratio, with lower sampling ratios favoring more similar molecules (Figure S1). This demonstrated the effectiveness of geometrically weighted sampling in tailoring the search scope. Further explanation of how this parameter facilitates the sampling process can be found below in the Dynamic sampling section.

### 2.4 Ligand Pose Alignment Reduces Docking Load

After generating a new ligand candidate, RosettaAMRLD aligns the structure of the proposed ligand to the previous one within the binding pocket. This three-dimensional alignment process leverages the structural similarities between the newly proposed ligand and its predecessor to expedite the placement of the new ligand, avoiding the computationally expensive process of full docking. Specifically, we use Open3DAlign in RDKit to map atoms from the prior ligand to the new one.^26,29^ Once the atom mapping is completed, Rosetta generates 200 rotamers for the new ligand and superimposes each onto the prior ligand’s pose. The rotamer with the lowest root-mean-square deviation (RMSD) based on the atom mapping is chosen to proceed to the next step. This alignment step approximates the pose that would result from a coarse-grained docking process, allowing for rapid initial placement of the ligand in the binding pocket. This streamlined approach saves computational resources by reducing the need for full docking calculations, while still ensuring that the new ligand is properly positioned for detailed refinement in the subsequent high-resolution docking.

### 2.5 Ligand Evaluation Through RosettaLigand Flexible Docking

After pose alignment, the high-resolution docking and final minimization steps from the standard RosettaLigand docking protocol are employed to refine the new pose.^21,22^ The high-resolution docking step employs an MCM algorithm to perform all-atom refinement of the ligand and side chains at the interface. The final minimization step optimizes the structure to a local minimum, allowing for minor backbone flexibility. Over the iterative design process, the protein structure accumulates local refinements based on similar poses, and therefore the protocol reduces the need for time-consuming complete docking at each iteration. To ensure the ligand remains appropriately positioned in the binding pocket, a filter is applied after docking to reject any poses where the ligand’s center of mass is located outside the pocket. This can happen sometimes when a bulky entity is proposed for a small or shallow pocket. The location filter prevents the generation of poses that are incompatible with the intended binding site.

RosettaAMRLD allows users to apply ligand-based filters before docking, such as molecular weight, number of rotatable bonds, or predicted log P values. These filters can be customized in the XML script and help to eliminate undesirable candidates early in the process, allowing for more targeted ligand optimization.

After final minimization, the proposed pose is scored following the RosettaLigand standard protocol, and the interface energy (in Rosetta energy units, REU) between the protein and the ligand is collected. Ligand efficiency (LE) is a commonly pursued metric in drug discovery.^30^ Especially at the early stages of discovery, a small and efficient hit allows further optimization into lead molecules without excessive molecular complexity. For this purpose, we calculate the design score *LE*_2_(*REU*) as normalized interface energy:^31^

$$LE_{2}\left(REU\right)=\frac{E}{\sqrt[{2}]{N_{HA}}}$$

where *E* is the interface energy and *N_HA_* is the number of non-hydrogen atoms or heavy atoms. This ″softened” measure of ligand efficiency avoids biases toward either excessively large or small molecules, ensuring a balanced evaluation of binding potential. If users prefer, they can switch to raw interface energy scoring in the protocol XML script, especially when the design goal involves larger molecules. We use *LE*_2_(*REU*) to evaluate and compare molecules throughout this paper, including the docking scores in the benchmarks.

### 2.6 Metropolis Criterion Prevents Trapping in Local Minima

In each Monte Carlo iteration, the newly proposed molecule is accepted or rejected based on its design score (*LE*_2_(*REU*)) relative to the current molecule. The probability of acceptance follows the Metropolis criterion,^32^ expressed as

$$p=\min\left(1,e^{-\Delta LE_{2}\left(REU\right)/kT}\right)$$

where Δ*LE*_2_(*REU*) is the difference in design score between the new molecule and the current one, *kT* is the scaling factor for the simulation temperature controlling the likelihood of accepting worse-scoring molecules. If the new molecule has a better design score, it is always accepted. If the new molecule has a worse design score, it is accepted with a probability of less than 1. The acceptance probability decreases as the score difference increases. The Metropolis criterion introduces an element of controlled randomness, which allows the system to occasionally accept worse-scoring molecules, preventing the algorithm from getting trapped in local minima and ensuring a more thorough exploration of the molecular space.

### 2.7 Dynamic Sampling Allows Adaptive Step Size for Efficient Chemical Space Exploration

Dynamic sampling in RosettaAMRLD allows for adaptive control over the exploration of chemical space by modulating the “step size” of ligand generation during Monte Carlo iterations. The step size, in this context, refers to the structural similarity between successive ligands. Selecting an appropriate step size is crucial—small step sizes may cause the search to get “trapped” in local minima, while large step sizes may lead to inefficient exploration of the chemical space. Here, we control the step size during ligand generation by varying the sampling ratio which dictates how likely the system is to select highly similar or dissimilar molecules. This approach reduces the number of docking evaluations required, as step size is adjusted before docking evaluation rather than through modifying the temperature in the Metropolis criterion in postgeneration steps. A smaller sampling ratio biases the algorithm toward selecting more similar molecules, resulting in smaller step sizes and more refined exploration of the local chemical neighborhood. Conversely, a larger sampling ratio increases the likelihood of selecting less similar molecules, facilitating broader exploration of more distant regions of the chemical space.

We tested the sampling performance under the MCM framework and measured the average step size of 100 design routes at different sampling ratios (Figure S2a). The step size was measured as the Tanimoto similarity between consecutive accepted ligands, with the average taken for each route. As expected, lower sampling ratios resulted in smaller step sizes (higher similarity between successive ligands), while higher sampling ratios led to larger step sizes, confirming that the sampling ratio directly controls the step size within the search.

We further analyzed how step size affected the diversity of the final output molecules. With independent routes all initialized from the same molecule, a larger step size allows each route to traverse a greater distance away from the starting point and from each other. Therefore, we hypothesized that a larger step size can increase the output diversity. To verify this hypothesis, we collected the output molecule from each design route and measured the diversity of the outputs by calculating the maximum Tanimoto similarity between each molecule and the rest of the set. Outputs are more diverse (have maximum similarities closer to zero) at higher sampling ratios, or larger step sizes, while at lower sampling ratios the outputs are more correlated (Figure S2c).

An efficient sampling strategy should be able to carefully examine “low energy regions” while also exploring a wide and diverse chemical space. In practice, the energy landscape is complex and a full exploration of the entire chemical space is infeasible with limited steps. We implemented a dynamic sampling strategy where the step size, or the sampling ratio, is adaptive to the acceptance status of the previous Monte Carlo iteration. Specifically, the user can set a sampling ratio range [*m*,*M*] and a constant rate of increase *s*. During the iterative process, the sampling ratio increases by *s* if the previous Monte Carlo iteration is rejected and reset to *m* if otherwise. Continuous rejections would cause a linear increase in the sampling ratio until reaches *M*, after which the sampling ratio is capped at *M* until the next Monte Carlo acceptance. This dynamic sampling strategy effectively adjusts the step size to the characteristics of the energy landscape as sampling progresses. For example, when the search is trapped in a local minimum where the neighborhood is mostly high-energy regions, the step size increases at every rejection until it escapes the neighborhood. Similarly, as the search proceeds to a new neighborhood after a Monte Carlo acceptance, it immediately resets to a smaller step size to ensure a detailed examination of the new neighborhood. Such a dynamic strategy can lead to faster convergence and a more efficient exploration of the chemical space.

We tested two dynamic sampling ranges, one with smaller sampling ratio thresholds and one with larger thresholds. Besides the general trend observed in average step size and output diversity with constant sampling ratio, the dynamic sampling trials maintained an average step size closer to the minimum for local refinement as shown in Figure S2b, while still delivering output diversity comparable to the broader search settings as shown in Figure S2d, implying that substantial movement has been taken occasionally to escape suboptimal regions. These results support that dynamic sampling improves the balance between local refinement and global exploration.

Sometimes, a fixed small step size is desired. For example, in a lead optimization case the sampling should scrutinize the local neighborhood. Another example is after the exploration phase and before a final output is proposed. It is preferable to perform a “lead-optimization-like” sampling stage to identify a local minimum as the final output. For this purpose, dynamic sampling can be switched off at later iterations to enable sampling with a fixed and small step size.

### 2.8 Two-Phase Optimization Strategy Enhances Exploration and Convergence to Minima

We adopted a “refinement after exploration” optimization strategy for all the production runs. Based on our investigation of the sampling ratio in Section 2.7, we find dynamic sampling with a minimum threshold of 0.01 and a maximum threshold of 0.2 an optimal setting in all tested protein targets to explore a diverse subspace as shown in Figure S2d. Therefore, we use it as the default setting for the first phase of the optimization. Since the minimum threshold is set to a moderate step size, we do not expect to fully explore every low-energy subspace on the route. Instead, we focus on identifying promising trajectories that cover a broad range of chemical diversity. Once the exploration phase concludes, the sampling ratio is adjusted to 0.0001, a small enough step size for most regions in the Enamine REAL space, and the search transition into the refinement phase, with dynamic sampling turned off. This shift ensures that the low-energy subspace reached at the end of the exploration phase is thoroughly examined and can rest to a local minimum.

Figure S3 shows example energy profiles of the optimization process in each protein target. The top ten design routes by *LE*_2_(*REU*) score were collected and the score at each accepted Monte Carlo iteration was plotted against the iteration number. The best-scoring route was highlighted in each protein case. A general downward trend was observed across all three targets, indicating successful energy minimization throughout the optimization process. The noticeable energy stabilization during the refinement phase further confirms the efficiency of the algorithm in converging to energy minima. Occasional increases in energy were also observed, particularly in the exploration phase, reflecting the Metropolis criterion’s ability to accept higher-energy states with a probability based on the system’s temperature. These occasional uphill moves allow the method to escape local minima, increasing the likelihood of finding better solutions.

We then analyzed the sampling scope of the two phases, measured by the similarity of the accepted molecules to the first accepted molecule in that phase. The similarity distributions of the top 10 design routes for all three targets are shown in Figure S4. The exploration phase exhibits lower similarity peaks, indicating that farther distance from the starting point was sampled with high frequency. In contrast, the refinement phase shows higher similarity peaks, reflecting a search within a more limited range. This difference demonstrates the effectiveness of the adopted strategy: the exploration phase identifies diverse, promising regions of chemical space, while the refinement phase focuses on optimizing these regions to converge on energetically favorable designs.

### 2.9 RosettaAMRLD Optimizes Ligands to Active-like Docking Scores Across Diverse Targets

We benchmarked RosettaAMRLD with 3 targets of different protein types: TrmD (enzyme), CDK2 (kinase), and OX1R (G-protein coupled receptor). All three targets have known binders and at least one available crystal structure, making them ideal candidates for validating the effectiveness of RosettaAMRLD. The combinatorial library used in the benchmark was the Enamine REAL space,^23^ which at the time contained over 30 billion synthetically accessible molecules generated through 290 reaction types and 2.1 million fragments. As a performance baseline, we constructed a set of 100 random molecules from the Enamine REAL space and docked them into each target structure. We then selected a poorly scored molecule from this random set for each protein target to initialize the RosettaAMRLD design process. By starting from a high-energy point in the chemical space, we aim to test the method’s ability to optimize ligands in a realistic setting where little is known about actual binders. Because of the stochastic nature of our method, we performed 100 runs for every protein complex and every setting to measure an average performance. Each run develops its search route independently and outputs a single best molecule from its route.

We evaluated the proposed molecules from a single round of RosettaAMRLD and compared them to the random baseline and known actives based on their predicted binding affinity indicated by the docking score. We redocked all the successful designs using the full RosettaLigand protocol and calculated a normalized interaction energy in the same way as the *LE*_2_(*REU*) score. Representative top-scoring molecules from AMRLD, known actives, and random sampling are shown in Figures S5–S7 for TrmD, CDK2, and OX1R, respectively. Figure 3 shows the docking score distributions for all three targets. To compare the distributions, we performed Student *t* test under the null hypothesis that the means of the two distributions are equal. Figure S8 shows a heatmap of t-statistics indicating the direction of the comparisons, with significance levels annotated based on corresponding *p* values. The AMRLD designs show a clear improvement over the random baseline (*p* < 0.0001), demonstrating that AMRLD can optimize low-scoring, random compounds to an extent that approaches the best-performing molecules from the random set. In the TrmD and CDK2 targets, only the upper quartile of AMRLD-designed molecule score as well as the known actives, which is likely due to actives in these cases being highly optimized from previous drug discovery efforts. In the OX1R case—where the actives originate from more general screening assays—the AMRLD designs score comparably to the actives (*p* > 0.05).

**Figure 3:**
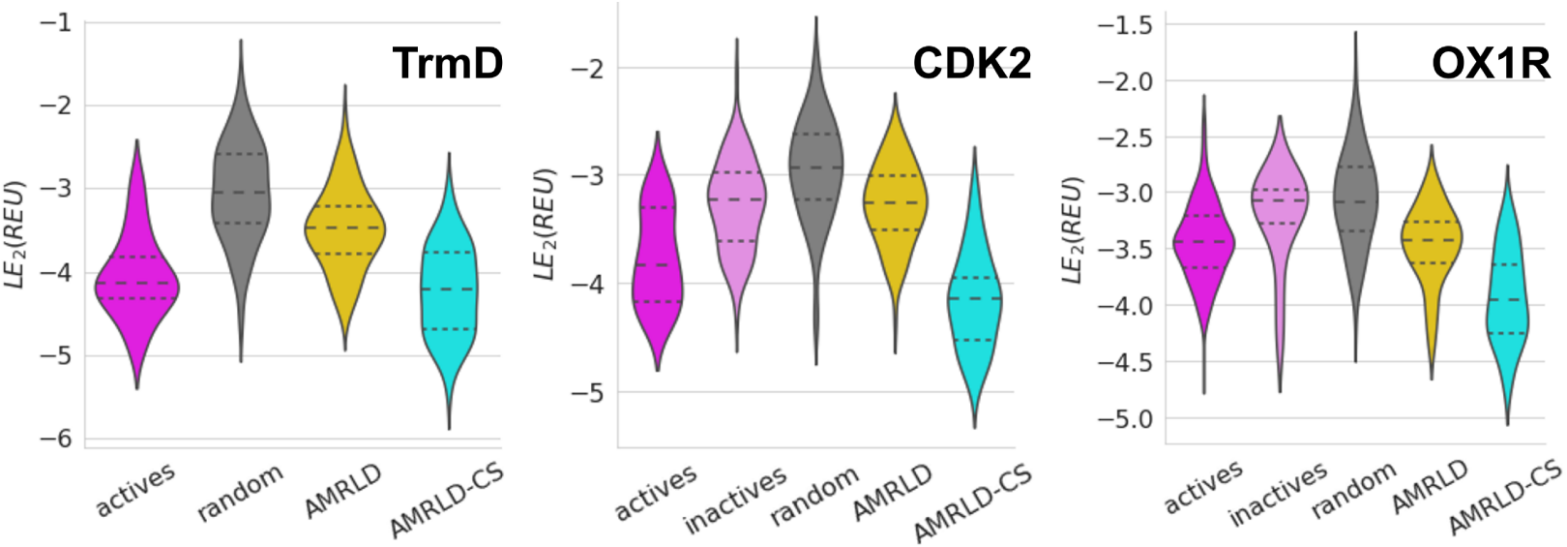
RosettaLigand docking score distribution for TrmD, CDK2, and OX1R. For each target, RosettaAMRLD (yellow) produced molecules with docking scores significantly better than those of random baselines (gray). RosettaAMRLD-CS (cyan) generated molecules with docking scores comparable to that of known actives (magenta) and better than the scores of known inactives (pink).

To provide an orthogonal evaluation of binding affinity, we rescored all AMRLD designs, random baselines, and known actives using GNINA’s convolutional neural network (CNN) scoring model and calculated *LE*_2_(*GNINA*) from the score term CNNaffinity.^33^ The resulting score distributions are shown in Figure S9 and the Student *t* test results are shown in Figure S10. Consistent with the RosettaLigand evaluation, AMRLD designs outperform the random baseline for TrmD (*p* < 0.05) and OX1R (*p* < 0.01). However, for CDK2, the performance is no different from random sampling (*p* > 0.05). When comparing to known actives, the GNINA scores agree with RosettaLigand that the actives are generally better than AMRLD designs for TrmD and CDK2 (*p* < 0.0001), while showing comparable scores for OX1R (*p* > 0.05).

### 2.10 Cascaded Sampling Further Enhances the Optimization Efficiency

As the size of combinatorial libraries continues to grow, so does the number of steps necessary to explore the chemical space. Different starting positions also lead to varied lengths of design routes to converge to minima. Yet increasing the number of Monte Carlo iterations proportionally increases the computation time. We hypothesized that further improvements to RosettaAMRLD results could be made by extending the promising routes through multiple rounds of optimization and evaluating a broader space. A similar approach in Rosetta has proven successful in improving the efficiency of sampling protein conformational space.^34^

In practice, the RosettaAMRLD protocol can be run on a computing cluster, where multiple design routes proceed independently and in parallel. Since MCM is a stochastic process, not all routes can identify an energetically favored region within the limited number of Monte Carlo iterations. The cascaded sampling workflow runs a number of RosettaAMRLD protocols in parallel, extending only the best-performing design routes (Figure 4). After each round of RosettaAMRLD optimization, the output ligands are ranked based on their design scores. A diverse subset of top-performing ligands is then selected as the starting point for the next round of optimization, allowing only the best design routes to continue. This selective extension of high-quality routes saves computational resources by focusing on ligands with the greatest potential.

**Figure 4:**
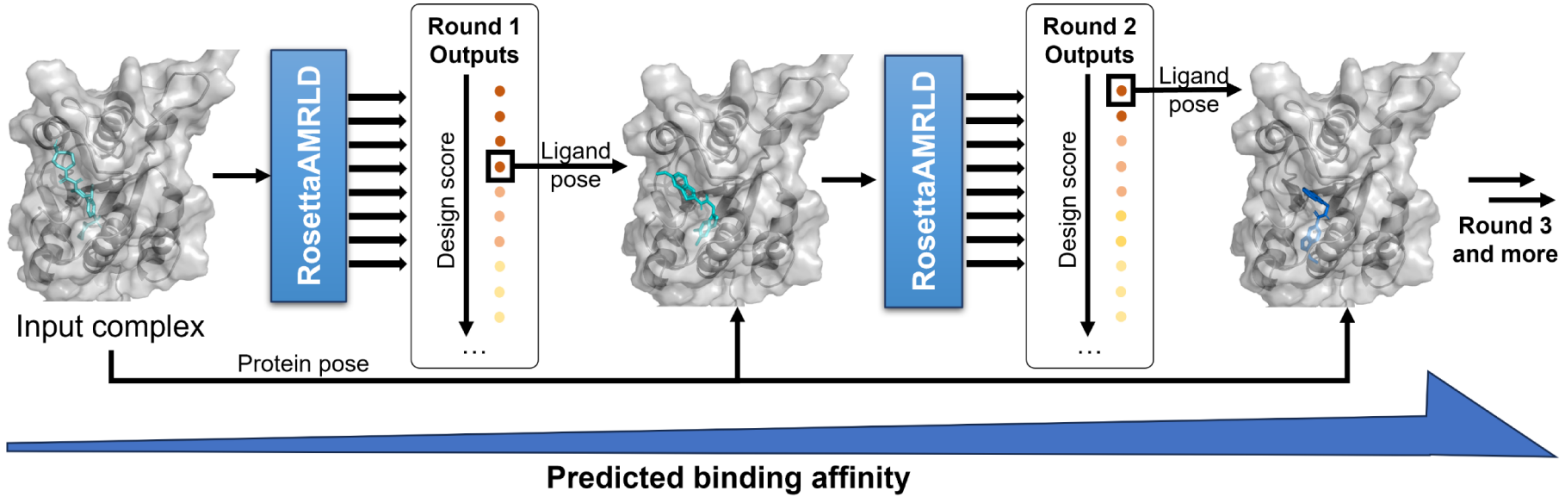
Cascaded sampling workflow.

Additionally, the workflow also helps to escape from local minima. Since the protocol includes a flexible high-resolution docking stage, the side chains are optimized at every iteration to better fit the current ligand. By transferring only the ligand pose to the next round and redesigning in the initial protein pose, the new complex stands in an unoptimized energy landscape, therefore having a better chance to escape the local neighborhood.

We applied multiround optimization with RosettaAMRLD (AMRLD-CS) to our benchmark set. For each additional round, a ligand from the top five designs in the previous round was manually chosen as the current round’s starting entity, and the ligand pose was transferred into the original protein structure to form the new complex. Each round used the standard setting and developed 100 independent routes from the starting entity. The effect of the cascaded sampling workflow is evident from the distributions in Figure 3 and the statistical comparisons in Figure S8, where we redocked the proposed molecules after three rounds of optimization. In all three targets, AMRLD-CS further improves the upper quartile of single-round molecules and consistently outperforms random molecules (*p* < 0.0001). For TrmD, the AMRLD-CS designs are similarly good to the known actives (*p* > 0.05), while for CDK2 and OX1R, the designs even exceed the known actives (*p* < 0.01 for CDK2 and *p* < 0.0001 for OX1R). Top-scoring designs from AMRLD-CS are shown in Figure S5–S7.

We also assessed the AMRLD-CS designs with GNINA as shown in Figure S9 and the corresponding statistical tests as shown in Figure S10. Compared to single-round AMRLD, the cascaded optimization process leads to score improvements in TrmD and CDK2, significantly exceeding random sampling (*p* < 0.0001). Although none of the protein targets have AMRLD-CS designs predicted to exceed actives, GNINA scores the AMRLD-CS designs matching the actives for CDK2 (*p* > 0.05). Interestingly, the score distributions for OX1R show substantial overlap and the *t* test results indicate no significant difference between AMRLD-CS designs and known actives as well as random baselines (*p* > 0.05).

Additionally, we compared AMRLD-CS to a straightforward extension of the standard AMRLD protocol, where we tripled the number of Monte Carlo iterations (Figure S11). While increasing Monte Carlo iterations provides some improvements, it fails to match the performance of AMRLD-CS. This suggests that selectively propagating top-performing ligands to multiround optimization is a more efficient strategy to search the chemical space than merely extending the sampling size of individual design routes. Collectively, these results demonstrated the value of the cascaded sampling workflow in exploring larger regions of the ultralarge library, ultimately identifying more robust local minima and higher-quality ligand designs.

### 2.11 RosettaAMRLD Generates Novel and Diverse Molecules

One of the primary goals of *de novo* drug design is to generate novel molecules that are structurally distinct from known active compounds. To assess the novelty and diversity of the ligands produced by RosettaAMRLD, we collected the designs from the last round of AMRLD-CS and evaluated their similarity to known actives, as well as their scaffold diversity across the three protein targets. Figure 5 shows the similarities between the AMRLD-CS designs and the known actives, with the random set similarities as a comparison. The distributions were plotted as the highest Tanimoto similarity between each design/random molecule and all actives. In all three protein targets, all the designs are novel molecules not present in the actives. In fact, the designs are quite distinct from the known actives, with the majority around 0.25–0.35 Tanimoto similarity. This is not surprising since no active is involved in any of the optimization processes and the designs originated from random molecules which are also very different from the actives. Nevertheless, the designs have a higher similarity to the actives than the random set, indicating that some structural features relevant to binding activity may be recaptured in the designs. We also calculated the percentage of unique Bemis-Murcko scaffolds^35^ in each individual set of molecules as a scaffold diversity metric for both the actives and the designs. The RosettaAMRLD designs showed high scaffold diversity, with over 90% unique scaffolds for TrmD and OX1R, and 81.3% for CDK2. In comparison, the active set of CDK2 displayed a scaffold diversity of 46.2%, indicating a more focused chemical space. This relatively low diversity among the actives again suggests that these active molecules have undergone extensive optimization in previous drug discovery campaigns, leading to a refined set of highly optimized compounds. This explains why the CDK2 designs have a relatively narrow and low similarity distribution to the active set. OX1R, on the other hand, has a diverse active set from generic screening assays, and therefore the designs have a relatively higher range of similarities to the actives. Overall, the high scaffold diversity in RosettaAMRLD designs indicates that the method explores a wider range of chemical space, making it well-suited for discovering novel chemotypes and scaffolds that can serve as starting points for further lead optimization.

**Figure 5:**
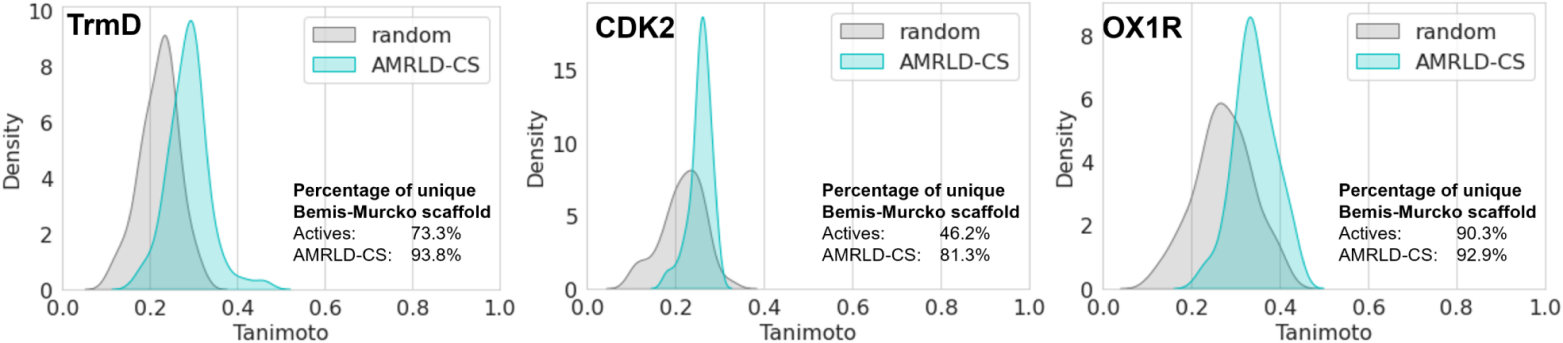
Similarity to known actives and scaffold diversity of AMRLD-CS designs. The Tanimoto similarity distributions between the AMRLD-CS designs (blue) and random molecules (gray) against the known actives are shown for TrmD, CDK2, and OX1R. The percentage of unique Bemis-Murcko scaffolds highlights the scaffold diversity in the AMRLD-CS designs.

### 2.12 RosettaAMRLD Designs Capture Active-like Binding Poses and Interactions

To further evaluate the novel designs as potential hits, we visually inspected the predicted binding poses of the top-scoring molecules from the AMRLD-CS runs and compared those to the crystal structures of corresponding protein targets. An example from each target is shown in Figure 6a–c where the design complex is overlaid onto the experimental structure with highlighted polar interactions. In all three targets, the top designs were able to capture similar interactions as the known actives. For TrmD and CDK2, the top designs share a similar pose as the actives, and with novel scaffolds. For OX1R, the binding pocket is relatively large and spherical, so we observe multiple different binding poses among the top designs.

**Figure 6:**
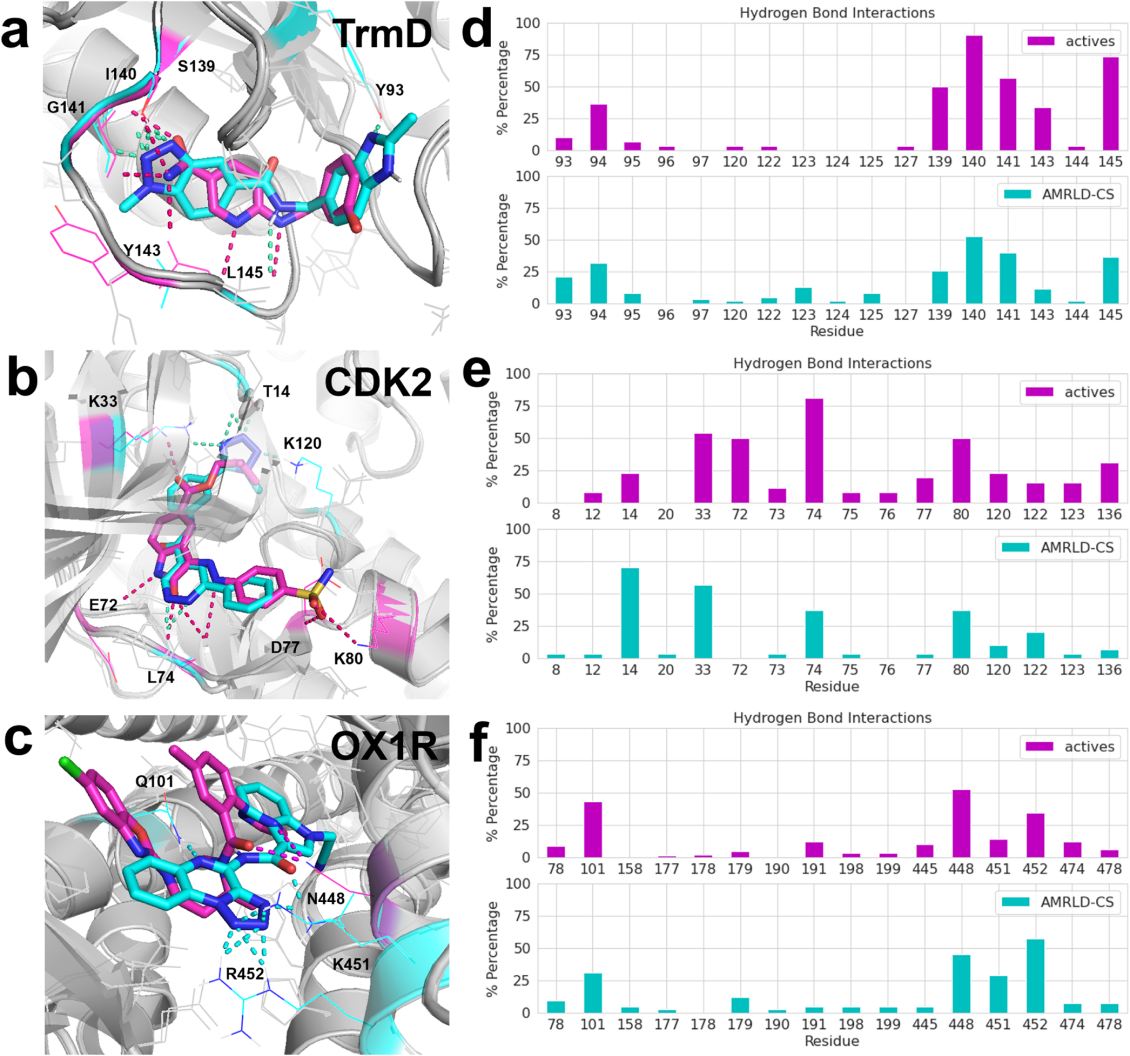
Predicted binding poses and hydrogen bond interaction analysis of AMRLD-CS designs compared to known actives. (a–c) Structural overlays of AMRLD-CS designs (cyan) with experimental structures (magenta) for TrmD (PDB 4YQ4), CDK2 (PDB 4FKW), and OX1R (PDB 4ZJ8), highlighting key polar interactions (dashed lines). (d–f) Protein–ligand interaction fingerprints generated by MOE for each target. The bar plots show the percentage of interactions found in actives (magenta) versus AMRLD-CS designs (cyan).

We then generated the protein–ligand interaction fingerprints (PLIFs) using the Molecular Operating Environment (MOE)^36^ for the redocked designs and known actives. The distributions of hydrogen bond interactions for each protein target are plotted in Figure 6d–f. In TrmD, the major interaction sites (residues interacting with over 50% of the actives) were recaptured in 20–50% of the designs. For CDK2, the major interaction sites were recaptured in 30–60% of the designs, except for residue E72, where none of the CDK2 designs capture the interaction. On the other hand, over 60% of the designs interact with residue T14, a site less frequently targeted by the known actives. This noticeable difference in the distribution of interaction sites between CDK2 designs and known actives can again be attributed to the nature of a focused set of highly optimized actives (46.2% scaffold diversity as shown in Figure 5). We observed a general trend that the actives of CDK2 and TrmD form more interactions with the pocket than our designs from the generic library. The repeating scaffolds and analogs in these actives contribute to the relatively high percentage (70–80%) of interactions at certain residues, both of which are characteristics of lead series. These lead molecules have gone through extensive optimizations by medicinal chemists to maximize their interactions with the target pocket. A generic library like the Enamine REAL space may not include the highly specialized structures that support such dense networks of coordinated interactions. It is important to note that this limitation does not imply that AMRLD is unable to identify or optimize such interactions but rather reflects the constraints imposed by the search space. When compared to the actives from a generic screening, like the OX1R case, the designs have a similar distribution of interaction sites, with the most-found interaction site present in around or below 50% of the molecules. Interestingly, in all three targets, some of the designed ligands introduced novel interaction sites that were not observed in the known actives, potentially offering new avenues for drug development.

### 2.13 Distribution of Physicochemical Properties

We compared the physicochemical properties of the AMRLD-CS designs, known actives, and random molecules for the three protein targets based on four key descriptors: molecular weight, number of hydrogen bond acceptors, number of hydrogen bond donors, and log P (Figure 7). The properties of the designs are largely decided by the combinatorial library, as can be seen from the overlaps between the designs and random molecule distributions. However, structural aspects from the protein pocket still have effects on the output molecules in these properties. For example, the molecular weight distributions of the library molecules are mostly within the 300–450 Da range. For the design molecules, we observe a slight left shift in smaller pockets (TrmD) and a right shift in bigger pockets (OX1R). Interestingly, the distributions of the number of hydrogen acceptors are noticeably higher in the designs than in the actives and random molecules. Since the random set is representative of the library and does not exhibit this enrichment, we attribute the bias to the scoring function, which may favor additional polar interactions during the docking process.

**Figure 7:**
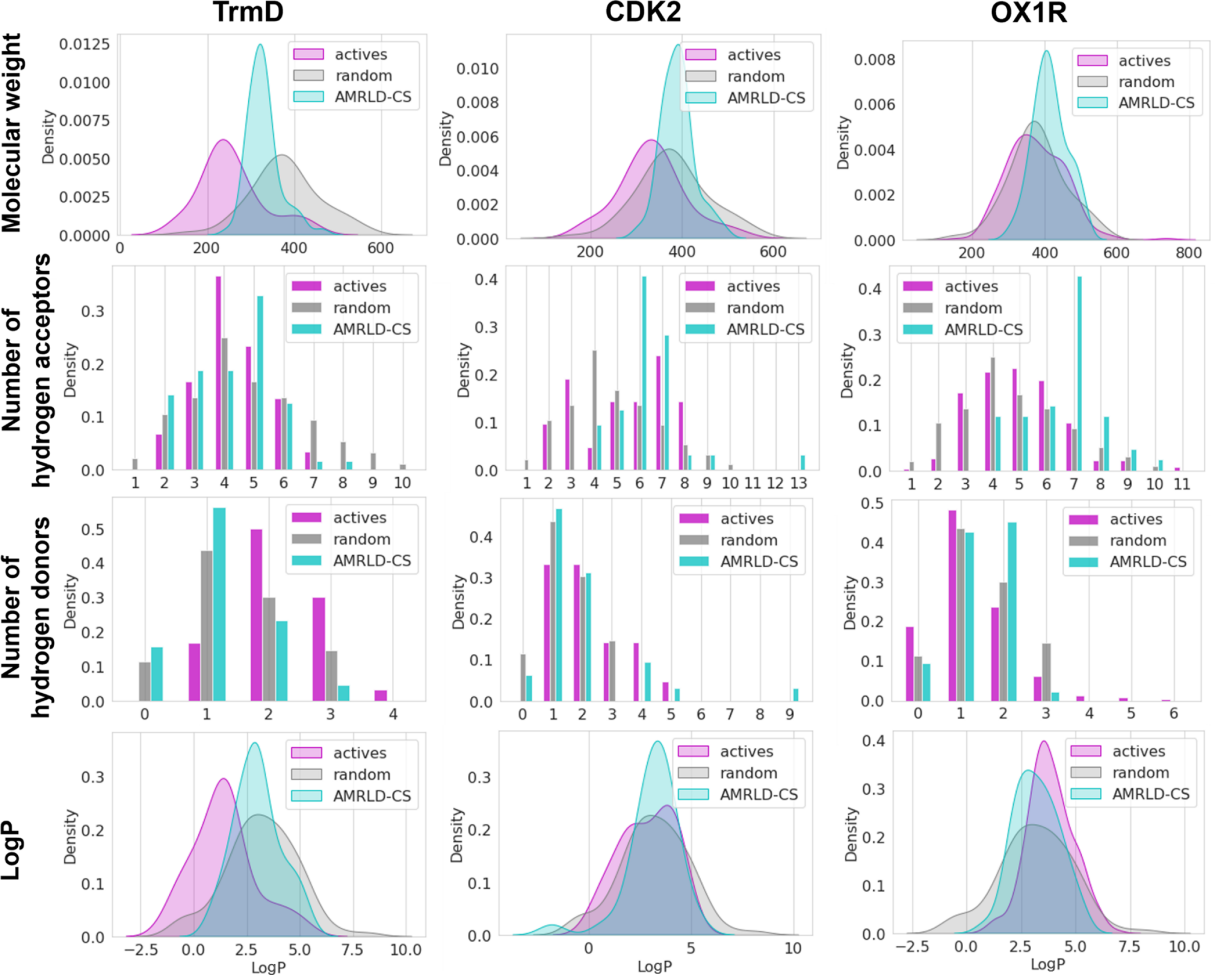
Comparison of physicochemical properties between AMRLD-CS designs (cyan), known actives (magenta), and random molecules (gray) for three protein targets. The distributions of molecular weight, hydrogen bond acceptors, hydrogen bond donors, and Log P are shown. A Log P bandpass filter was included in the protocol which focuses on molecules with 0 ≤ log P ≤ 5. Molecules outside this range are penalized in score and rejected if log P < −1 or log P > 8.

In practice, the user can customize the database or add physicochemical property filters to better guide RosettaAMRLD toward the set of molecules with desired properties. We did not prefilter the reactants from the Enamine REAL Space to fit each protein target since we assumed no prior information was known about the target’s binders and assessed the method’s performance when provided with only the target structure. We did include a log P bandpass filter in the protocol XML which focuses on molecules with 0 ≤ log P ≤ 5. Molecules outside this range are penalized in score and rejected if log P < −1 or log P > 8. This has successfully limited the outputs to those with log P < 6, showcasing how the physicochemical filters can be applied to guide the design. Additionally, we included a Synthetic Accessibility Score (SAscore) filter to exclude overly complex molecules (SAscore >6) prior to docking.^18^ However, when working with make-on-demand libraries such as the Enamine REAL space—where all molecules are readily accessible—this filter may be somewhat redundant. Nevertheless, we retain this filter to support users who may apply RosettaAMRLD to customized libraries, where synthetic accessibility is not guaranteed.

### 2.14 RosettaAMRLD Promotes Scaffold Hopping in a Lead Optimization Scenario

A typical lead optimization effort involves identifying structural analogs of a lead and evaluating them through docking to select the next series of compounds for testing. To test RosettaAMRLD in such a scenario, we conducted a single-round optimization using the standard parameters, initiating from the native ligand of TrmD crystal structure (PDB ID: 4YQ9) as shown in Figure S12a. In parallel, we retrieved the top 100 most similar analogs from the Enamine REAL database using SmallWorld, with the same native ligand as the reference.^37^ All molecules were then docked with RosettaLigand, and their $LE_{2}\left(REU\right)$ score distributions were compared to each other, to those of known actives, and to AMRLD outputs initiated from a poorly scoring random compound, as shown in Figure S12b.

The results demonstrate that RosettaAMRLD under the standard setting has a consistent performance in optimizing the docking scores regardless of the starting point in the chemical space. When initiated from an active ligand, AMRLD achieved comparable or slightly improved docking scores relative to the random-start scenario. While similarity search analogs exhibited a better and narrower score distribution centered near the score of the reference ligand—as expected—their scaffold diversity was limited, with only 28% of them having unique Bemis-Murcko scaffolds. In contrast, AMRLD designs showed significantly greater scaffold exploration, with 88% of the output featuring unique scaffolds. The top 10 molecules by $LE_{2}\left(REU\right)$ from AMRLD and similarity search are shown in Figure S12c, d, respectively. This result suggests that RosettaAMRLD is particularly well-suited for scaffold hopping purposes in lead optimization efforts.

## 3 Discussion

We have shown the effectiveness of RosettaAMRLD in generating novel, synthetically accessible ligands that outperform random baselines and approach the performance of known actives in different types of protein targets. Compared to traditional structure-based virtual screening methods, RosettaAMRLD refrains from the need to evaluate the entire targeted chemical space and offers greater flexibility in searching for chemical entities of interest. This advantage allows RosettaAMRLD to better utilize the ultralarge combinatorial libraries and meet various design objectives including *de novo* design, lead optimization, scaffold hopping, etc. This approach also complements recent reaction-based frameworks for navigating ultralarge libraries, such as the Thompson Sampling method by Klarich et al.^19^ and the Monte Carlo Tree Search strategy by Swanson et al.^20^ RosettaAMRLD is distinguished by its underlying algorithm which is an MCM framework. Furthermore, unlike Klarich et al. where the method is compatible with either SB (docking) or LB (similarity) scoring, RosettaAMRLD integrates both docking scores and molecular similarity to guide sampling decisions. When compared to Swanson et al., which is an LB method and requires preexisting experimental data to train a property prediction model, our SB approach can be applied to novel targets with no prior binder, evaluating candidate molecules directly within the structural context. These differences highlight RosettaAMRLD’s novelty and potential in SB drug design.

The increasing availability of protein structures offers unprecedented opportunities for structure-based drug design. Our reported results used crystal structures to reduce the source of uncertainty from the protein structure. However, RosettaAMRLD is not limited to using experimental structures. Other sources of structure such as AlphaFold^9^ and homology models can all serve the purpose. Our docking evaluation uses RosettaLigand, which performs flexible docking with full-atom refinement of the ligand and side chains, as well as minor backbone flexibility, in each Monte Carlo iteration.^21,22^ To further account for protein conformational variability, users may initiate RosettaAMRLD from multiple conformations of the same protein as separate starting complexes—each conformation forms its own independent design route. Although RosettaAMRLD currently does not support an ensemble of protein–ligand complexes as the input, integration of ensemble-based design is an open direction for future development.

Additionally, RosettaAMRLD requires no information as to known actives or inactives. In benchmarking, the design routes were started from a computationally docked poorly scoring random molecule from the Enamine REAL space, meaning that there was hardly any useful interaction information that could be drawn from the initial complex. Starting the run with a molecule directly from the targeted chemical space also avoids potential “bad mapping” from the actives to the defined search space, which could result in loss of interaction information. This usually happens when the reference molecule is a well-engineered lead compound that is too specific to find analogs in a generic chemical library. Such lead compounds are also local minima in the defined space, making it challenging for the MCM algorithm to escape and explore efficiently.

We tested single-round RosettaAMRLD in a lead optimization scenario for TrmD, using a known active ligand as the starting point. The standard parameters we used serve as a baseline, and performance can be further enhanced through parameter fine-tuning and multiround optimization (cascaded sampling). However, we did not report multiround results in this context, as our preliminary testing for the lead optimization scenario indicated that the optimal parameter set and number of rounds are target-specific and in some cases lead-specific (when “bad mapping” arises, as mentioned above). While we are actively working to improve RosettaAMRLD for more robust sampling and streamlined user experience, we encourage users to experiment with different settings tailored to their specific design goals. For example, if the objective is to explore analogs of a lead molecule, users might consider turning off dynamic sampling and setting a smaller sampling ratio to bias local search. Additionally, a focused library that defines the lead’s neighborhood can be utilized. If the aim is to discover novel molecules or scaffold hopping, we suggest using scaffolds from the lead molecule to start the design process. Active scaffolds (without associated pendent moieties) are likely higher in energy, and therefore easier to escape from. It would be also easier to map a scaffold onto the generic chemical library than a very specific molecule.

The choice of RDKit Daylight-like fingerprints and the specific Tversky index for fragment ranking resulted from a series of evaluations on fingerprints and similarity metrics. We tested several alternatives including Morgan fingerprints—both ECFP (extended connectivity fingerprints) and FCFP (functional-class fingerprints)—as well as RDKit pattern fingerprints.^38^ However, Morgan fingerprints paired with Tversky substructure similarity often failed to rank fragment analogs intuitively. We also evaluated several Tversky indices with varied reference-fragment weights, and among all combinations tested, the pairing of RDKit Daylight-like fingerprints with Tversky (0.1 – reference, 0.9 – fragment) consistently produced the most reasonable rankings. As a result, the current implementation does not permit user-defined fingerprint or similarity settings. Nonetheless, our evaluation was not comprehensive, and we recognize the potential of other combinations. Future updates of RosettaAMRLD may include support for customizable fingerprints and similarity options.

Our current similarity-guided fragment selection compares the entire reference ligand to the fragment library using a Tversky substructure similarity metric. During early development of RosettaAMRLD, we also considered an alternative approach where individual fragments of the reference ligand were compared to those in the library. While intuitive, this approach presented several limitations. Molecules sampled along a design trajectory often fragmented at consistent positions, reducing opportunities for structural rearrangement. Additionally, transitions between products of reactions with differing numbers of components became more constrained. These challenges ultimately restricted exploration to narrower regions of chemical space. Our current implementation was developed to provide greater sampling flexibility and support transitions across diverse reactions, ultimately resulting in more desirable sampling distributions in a vast chemical space.

Effective sampling of the chemical landscape is also achieved by balancing local refinement and broader exploration during the sampling process. This is controlled in RosettaAMRLD by adapting the sampling ratio, which controls the step size during ligand generation. Using dynamic sampling, where the sampling ratio is adjusted based on the recent success in searching, helps to navigate different energy landscapes and improves sampling efficiency in a wider chemical space. We have found through testing a set of sampling parameters that have a robust and effective performance in all three benchmark targets. Yet the parameter space was not exhaustively explored. It is possible that alternative parameter configurations may further improve the outcome in specific targets. Similarly, our current approach of a two-phase half/half split optimization strategy for sampling ratio regimes has not been comprehensively optimized. Future work should evaluate the effect of different sampling ratio schedules to further enhance sampling efficiency.

Future studies will also investigate the impact of library size and density (the number of structural analogs within a defined subspace) on the choice of sampling ratio range. Our geometrically weighted sampler is highly dependent on the ranking of the similarities, rather than the similarity scores themselves. In dense chemical spaces, where structurally similar molecules are clustered together in large numbers, a significant difference in ranking may correspond to only minor differences in similarity. Consequently, a larger sampling ratio may be required to achieve the same step size as in a less dense library. In practice, chemical libraries are often unevenly distributed in terms of density. One potential solution is to develop an advanced sampler that adapts dynamically to local density, similar to how we account for database size in the current methodology. Alternatively, as commercial libraries continue to grow denser and larger, clustering methods could be employed to normalize densities, reducing the library into fixed-density subsets. Additional local optimizations could then focus on individual clusters with high potential.

The cascaded sampling workflow is a further approach to efficiently sample the chemical landscape. By selectively extending only the best-performing design routes after each optimization round, we can focus on promising ligands, thus improving efficiency. The workflow is semiautomated, providing flexibility for the user’s choice of promising routes. We encourage the user to visually inspect the top-scoring ligands between rounds and select a diverse set of molecules appropriate to the design purpose for further optimization. Although the intention of cascaded sampling is to expand the search scope, we sometimes observe convergence between extended routes as similar molecules to prior rounds are proposed in later rounds. Such convergence is a common characteristic of MCM methods and is indicative of the identification of robust local minima.

RosettaAMRLD has successfully leveraged structural information to generate novel designs with active-like binding poses and interactions. Without any prior knowledge of the active molecules’ configurations during the optimization process, the method has generated functionally relevant designs solely from structural information on the protein targets. The designs not only captured key interaction points found in known actives but also introduced new interaction sites, highlighting the ability to explore untapped areas of the binding pocket. Overall, RosettaAMRLD has proven capable of creating novel scaffolds while maintaining key active-like interactions, suggesting that these *de novo* designs hold promise as potential hits for further experimental evaluation.

While our benchmarking results demonstrate that RosettaAMRLD consistently outperforms random sampling and often approaches or even exceeds known actives, these conclusions are inherently tied to the scoring function used for evaluation. Our analysis using both RosettaLigand and GNINA scoring functions reveals discrepancies between the two scoring methods. For example, the multiround RosettaAMRLD designs are predicted to match or exceed known actives with RosettaLigand, but less so with GNINA. On the other hand, the overlapping score distributions for OX1R in Figure S9 reflect either limitations in GNINA’s ability to distinguish actives from decoys in specific protein targets, or that GNINA’s scoring criteria do not align with RosettaLigand docking evaluations. These results underscore a critical limitation in the field: current scoring functions are still imperfect proxies for true binding affinity and may bias performance evaluations. While novel sampling methods like RosettaAMRLD rely on the scoring function to validate the results, their actual success is often over- or underestimated. Nevertheless, RosettaAMRLD can be paired with any scoring function, including future, more accurate scoring methods. The concept behind its optimization process should be adaptable to any binding energy prediction method, so as protein–ligand binding affinity prediction methods improve, RosettaAMRLD optimization should improve likewise.

While the current study presents only *in silico* validation, we recognize that performance of the proposed molecules *in vitro/in vivo* may vary from the predicted binding affinity. Experimental testing remains the gold standard for assessing the real-world utility of novel sampling methods. Although we did not purchase or biologically evaluate the compounds for the benchmark targets here, RosettaAMRLD is actively being applied to other pharmaceutical targets as part of other ongoing works, where selected compounds are purchased and tested experimentally. These efforts will provide prospective validation of the method and help guide further improvements to our sampling strategies.

## 4 Conclusion

RosettaAMRLD provides a powerful and innovative tool for structure-based *de novo* drug design, combining the sophisticated MCM framework in Rosetta with reaction-based sampling to prioritize synthetically accessible molecules from ultralarge combinatorial libraries. Importantly, RosettaAMRLD ensures that every proposed molecule is synthetically accessible, an often overlooked aspect in many computational design methods. By closing the gap between computational design and experimental validation, our method serves as a practical tool for drug discovery, providing compounds that are not only theoretically promising but also experimentally actionable. Our approach ensures efficient exploration of vast chemical spaces, leading to high-quality, novel ligand proposals. The adaptive sampling strategy and customizable XML protocol allow the user to tailor the search process to a specific design purpose. RosettaAMRLD balances broad chemical exploration with detailed local refinement, leading to optimized ligand designs that exhibit favorable physicochemical properties. This capability, along with the method’s efficiency in handling ultralarge libraries, positions RosettaAMRLD as a strong alternative to traditional SBVS approaches, especially in early stage drug discovery where the rapid identification of hit compounds is critical.

The application of RosettaAMRLD to three distinct protein targets—TrmD, CDK2, and OX1R—demonstrates its versatility and effectiveness. The method consistently produced novel ligands with docking scores that surpassed those of randomly selected molecules, and with the cascaded sampling workflow, rivaled or exceeded the predicted binding affinity of known active compounds. The results across multiple protein targets confirm the method’s utility in generating promising molecules with diverse scaffolds while maintaining key protein–ligand interactions. The method demonstrated great success in scenarios where no prior knowledge of active molecules exists. High-energy random scaffolds can be incrementally refined into lower-energy, biologically relevant structures. This makes RosettaAMRLD particularly useful for novel targets. Although in some cases, RosettaAMRLD designs did not capture as many structural interactions as a highly optimized lead series, the proposals still represent decent starting points for further optimization.

Our work focused on demonstrating the feasibility of RosettaAMRLD, while many aspects of it can still be improved. Future research will investigate additional adaptive sampling strategies to further improve sampling efficiency. It is also worth implementing effective simulated annealing algorithms to adjust the temperature in the metropolis criterion during the search process. More importantly, we would like to bring our approach to practical usage and validate the proposed molecules experimentally.

## 5 Methods

### 5.1 Protein Structure Preparation

We obtained high-quality crystal structures for each target from the Protein Data Bank (PDB):^39^ TrmD (PDB: 4YQ9), CDK2 (PDB: 4EK4), and OX1R (PDB: 4ZJ8).^40^ These structures were cleaned by removing all atoms except those of chain A proteins. The constrained relax protocol in Rosetta was then applied to the cleaned structures with the following flags: “-default_max_cycles 200 -relax:constrain_relax_to_start_coords -ramp_constraints false”.^41^ A total of 100 relaxed models were generated for each target, and the model with the best total score was selected for subsequent design runs. Binding pocket locations were defined based on the cocrystallized ligands, with the center of each pocket set at the coordinates of the ligand center, and a 7.5 Å radius defining the allowable range for ligand movement during optimization.

### 5.2 Benchmark Data Sets

The benchmark data sets for known binders and nonbinders were obtained from publicly available resources. For TrmD and CDK2, we used data sets from the Drug Design Data Resource,^42^ which were originally published by the Community Structure–Activity Resource.^43,44^ These data sets included 30 known binders for TrmD, and 26 binders and 35 nonbinders for CDK2. For OX1R, we sourced a high-throughput screening data set from the PubChem database,^45^ which contains 234 actives and 13 inactives.^46^ These molecules were docked with RosettaLigand standard docking protocol^22^ into the corresponding prepared target structure to obtain a normalized interaction energy for comparison to the RosettaAMRLD designs.

### 5.3 Combinatorial Library and Random Molecule Generation

The Enamine REAL Space we used for benchmarking is the 2023–01 release, comprising more than 30 billion molecules. We obtain it directly from Enamine under a nondisclosure agreement. The library is provided as 290 two-component or three-component reactions represented in SMILES Arbitrary Target Specification structure (SMARTS) and their corresponding reagents, a total of 2,131,807 synthons, in SMILES string format. The random baseline molecules were generated through two steps: 1) randomly select 100 reactions with replacement; 2) for each chosen reaction, randomly select one synthon for each of the reaction components without replacement. These random molecules were docked using the same RosettaLigand protocol as the known binders to obtain a normalized interaction energy.

### 5.4 Single-Round RosettaAMRLD Optimization

The initial molecules for single round optimization were chosen from the docked random molecules. For each benchmark target, the 100 random molecules were ranked by their normalized interaction energy LE_2_(REU) from the worst to the best. To ensure chemical diversity and avoid overly simplistic starting points, molecules composed solely of alkyl chains were excluded from consideration. The final molecules chosen as the starting points were among the top five worst-scoring molecules for each target, as shown in Table 1. The RosettaAMRLD protocol was set to run 2000 Monte Carlo iterations. Dynamic sampling was turned on for the first 1000 iterations, with minimum and maximum thresholds for sampling ratio set to 0.01 and 0.2, respectively. The step of increase after a rejected iteration is set to 0.002. After 1000 iterations, dynamic sampling is turned off and sampling ratio is fixed at 0.0001. Additional parameter settings can be found in the associated protocol capture in the Supporting Information. After optimization, the duplicates were removed and the unique designs were redocked with the same RosettaLigand protocol as the known binders to obtain a normalized interaction energy.

**Table 1:**
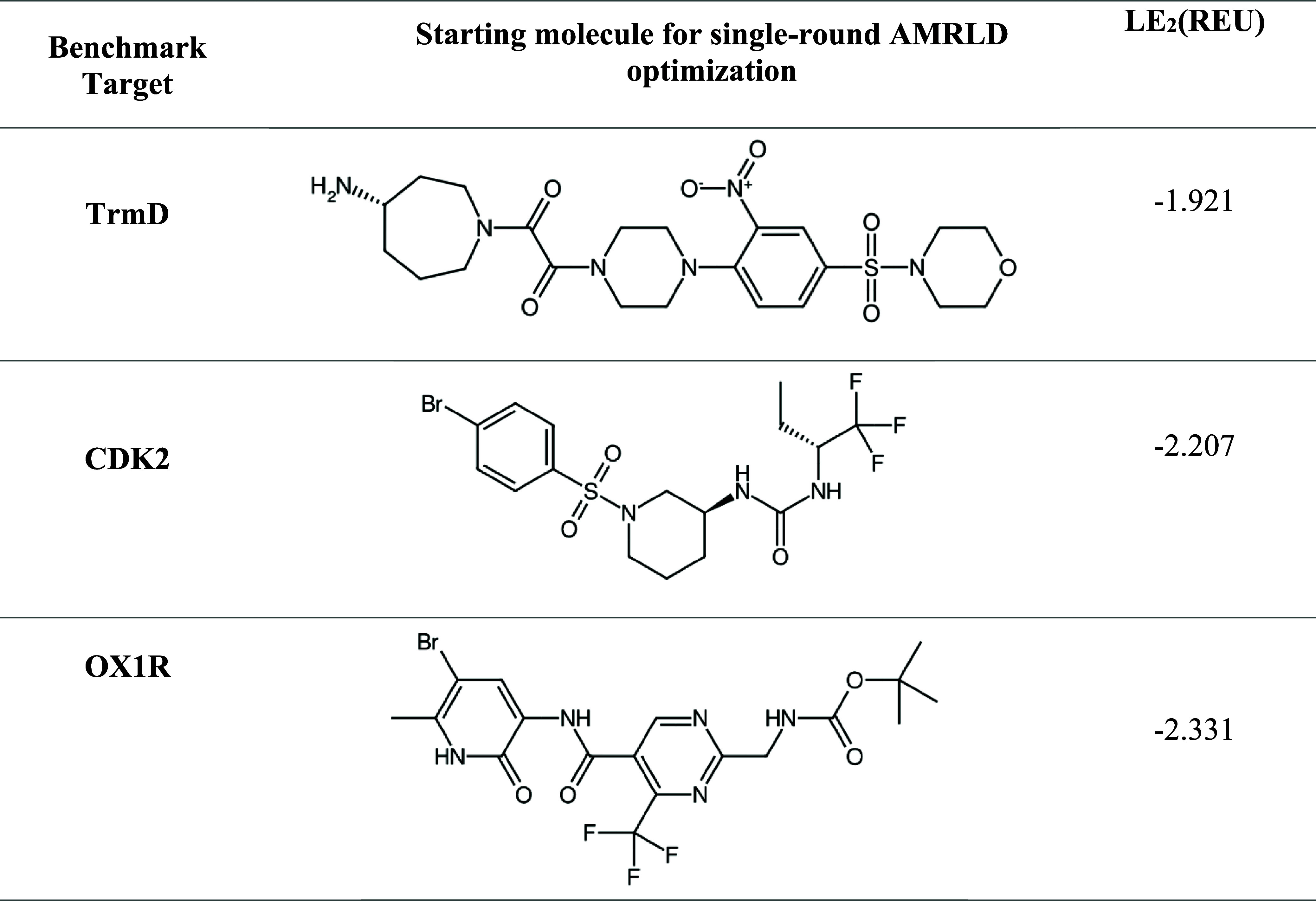
Initial Chemical Entity and Normalized Docking Score for AMRLD Single-Round Optimizations

### 5.5 Multiround RosettaAMRLD Optimization

The reported AMRLD-CS results were generated with a total of three rounds of optimization using the cascaded sampling workflow: starting from the single-round results, two more rounds were performed for the best-scoring designs. The starting molecule for each of the following rounds was one of the top 5 designs in the prior round (Figures S13–S15). Each additional round followed the same design setting as the single-round optimization. The reported AMRLD-CS results were generated from the third-round designs only.

### 5.6 Runtime

The production runs were conducted on the high-performance computing clusters at the Advanced Computing Center for Research and Education (ACCRE) at Vanderbilt University. A single run with 2000 Monte Carlo iterations and standard settings requires a single CPU 16–32 h to finish, depending on the size of the protein target.

## Supplementary Material





## Data Availability

Protein structures are available on the Protein Data Bank (PDB) with entry ID mentioned in the paper. Known actives and inactives are available through Drug Design Data Resource (TrmD and CDK2) and PubChem (OX1R). Access to the Enamine REAL space can be requested through Enamine Ltd. RosettaAMRLD is distributed as part of the Rosetta repository (https://github.com/RosettaCommons/rosetta). All code and scripts for RosettaAMRLD can be found under the following path in Rosetta: rosetta/source/src/protocols/drug_design/.

## Notes

Mathematical derivation of geometrically weighted sampling, additional benchmarking results, and example top-scoring molecules from cascaded sampling (PDF)
Protocol Capture: Example input and output files, Rosetta XML scripts and option files, postprocessing and data analysis scripts, instructions, and example commands (ZIP)
