# Supplementary material for: RosettaAMRLD: A Reaction-Driven Approach for Structure-Based Drug Design from Combinatorial Libraries with Monte Carlo Metropolis Algorithms
Source: J Chem Inf Model. 2025 Jun 11;65(12):5945–59. doi: 10.1021/acs.jcim.5c00497 (PMC12199295; doi:10.1021/acs.jcim.5c00497)
Supplement: Supplementary file 1 [file ci5c00497_si_001.pdf]

RosettaAMRLD: A reaction-driven approach for structure-based drug design from combinatorial libraries with Monte Carlo Metropolis algorithms

Yidan Tang[1], Rocco Moretti[1,2], Jens Meiler[1,2,3,4,5]**

[1]Department of Chemistry, Vanderbilt University, Nashville, TN, 37240, USA

[2]Center for Structural Biology, Vanderbilt University, Nashville, TN, 37240, USA

[3]Institute for Drug Discovery, Faculty of Mathematics and Informatics, Faculty of Chemistry and Mineralogy, University of Leipzig, Leipzig, 04103, Germany

[4]Center for Scalable Data Analytics and Artificial Intelligence ScaDS.AI and School of Embedded Composite Artificial Intelligence SECAI, Dresden/Leipzig, 04103, Germany

[5]Department of Pharmacology, Institute of Chemical Biology, Center for Applied Artificial Intelligence in Protein Dynamics, Vanderbilt University, Nashville, TN, 37240, USA

Corresponding Author

*Rocco Moretti rocco.moretti@vanderbilt.edu

*Jens Meiler jens@meilerlab.org

Supplementary Information

Section 1: Derivation of Geometrically Weighted Sampling

Given a sorted list of molecules of size N, a sampling ratio x, and a total normalized sampling weight of 1, we define $n = xN$ such that the sum of normalized sampling weights for the first n molecules in the sorted list is $1 - x$. Given this property holds for all consecutive subsets of the sorted list, the partial sums of the sampling weights follow a convergent geometric series with the first term $0 < r < 1$ and common ratio $1 - r$. According to the summation formula for any geometric series with first term a_1 and common ratio q

$$S_n = \frac{a_1(1 - q^n)}{1 - q}$$

We can express r in the form of sampling ratio x and list size N

$$1 - x = \frac{r\big(1 - (1 - r)^{xN}\big)}{1 - (1 - r)}$$

$$1 - x = 1 - (1 - r)^{xN}$$

$$x = (1 - r)^{xN}$$

$$r = 1 - \sqrt[xN]{x}$$

Therefore the k-th molecule in the geometric series has a weight of

$$a_k = (1 - \sqrt[xN]{x})(\sqrt[xN]{x})^{k-1}$$

Therefore, the "rate of decay" for the geometrically distributed weights down the sorted list is decided by the adjustable sampling ratio x and the number of molecules N in the list or the library.

For any geometric series with first term $a_1 = r$ and common ratio $q = 1 - r$, with infinitely many terms,

$$S_\infty = \frac{a_1}{1 - q} = \frac{r}{1 - (1 - r)} = 1$$

Therefore the geometric series converges towards 1 with infinite terms.

However, the list or library doesn't have infinitely many molecules. The real sum of sampling weight over all molecules can be expressed as a function of sampling ratio x,

$$S_N = \frac{a_1(1-q^N)}{1-q}$$

$$S_N = \frac{r(1-(1-r)^N)}{1-(1-r)}$$

$$S_N = 1-(1-r)^N$$

$$S_N = 1-(1-(1-\sqrt[xN]{x}))^N$$

$$S_N = 1-(\sqrt[xN]{x})^N$$

$$S_N = 1-\sqrt[x]{x}$$

Note that the final equation for S_N is independent of N. This function deviates from 1 as x increases. Thus our assumption of total weight approximates to 1 is only valid at a small sampling ratio x. Since the sampling ratio x is adjustable by the users, we recommend using $x \leq 0.25$ to hold the assumption of approximation up to two decimal places.

Section 2: Tversky index and Tanimoto similarity

The Tversky index and Tanimoto similarity are widely used metrics for quantifying the similarity between chemical structures, based on their molecular fingerprints.

The Tversky index is a generalized similarity measure that extends other metrics, including the Tanimoto similarity. For two molecules A and B, the Tversky index is defined as:

$$Tversky(A,B) = \frac{c}{c+\alpha(a-c)+\beta(b-c)}$$

where a and b are the number of features in A and B, respectively; c is the number of features shared by A and B; α and β are weighting factors that adjust the contributions of features unique to A and B, respectively. By adjusting α and β, the Tversky index can be customized to emphasize similarity to one molecule over the other. For instance, setting $\alpha = 0$ and $\beta = 1$ gives:

$$Tversky(\alpha = 0, \beta = 1) = \frac{c}{b}$$

This asymmetric measure entirely ignores the features unique to molecule A and calculates the similarity as the proportion of common features in molecule B. When the proportion equals one, molecule B is a substructure of molecule A. This makes it particularly useful for sampling fragment analogs and prioritizing the ones that retain key substructures of the reference molecule in RosettaAMRLD. To better rank various fragment analogs, we use $Tversky(\alpha = 0.1, \beta = 0.9)$ which sets a small weight on molecule A (the reference structure), as suggested by Daylight Chemical Information Systems, Inc. (https://www.daylight.com/dayhtml/doc/theory/theory.finger.html).

The Tanimoto similarity is a special case of Tversky index where $\alpha = \beta = 1$. In this case, the formula simplifies to:

$$Tanimoto(A, B) = Tversky(\alpha = 1, \beta = 1) = \frac{c}{a + b - c}$$

This symmetric measure calculates the proportion of shared features to the total unique features across A and B, making it suitable for comparing molecules of similar size. Therefore in RosettaAMRLD, the similarities between candidate products are calculated by Tanimoto similarity.

Section 3: Figures

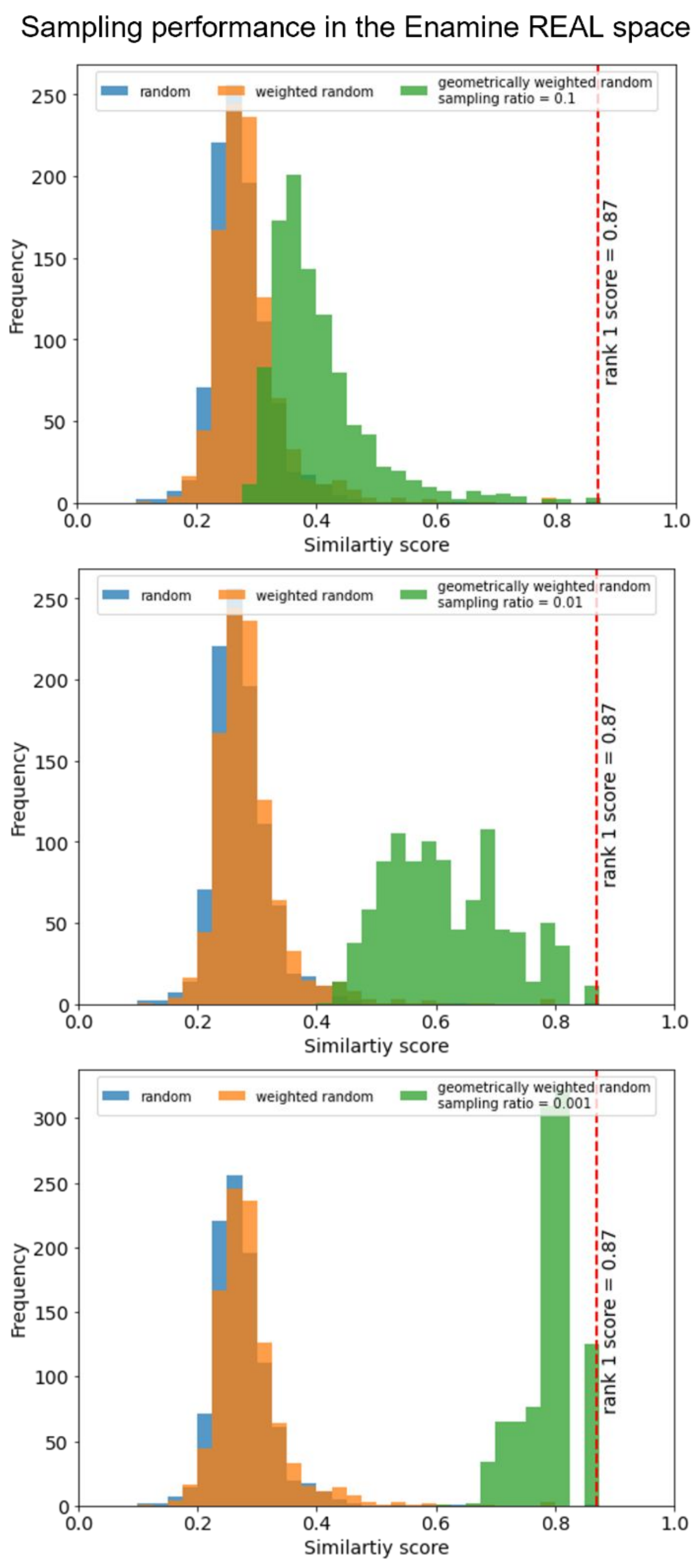

Figure S1. The performance of a geometrically weighted sampler (green) with a sampling ratio of 0.1/0.01/0.001 (top, middle and bottom panel) compared to a random sampler (blue) and a similarity-weighted sampler (orange). A known active of TrmD (PDB ID: 4YQ9) was used as the reference molecule. Based on the Tversky(0.1 – reference, 0.9 – fragment) similarity score with RDKitFingerprint, we sample the Enamine REAL space (2.1 million fragments) 1000 times with each sampler and plot the similarity score distributions of the samples. The similarity-weighted sampler is unable to bias the sampling towards the highly similar fragments and has the same sampling performance as a random sampler. The geometrically weighted sampler biases the sampling towards highly similar fragments, with the degree of bias adjustable through the sampling ratio.

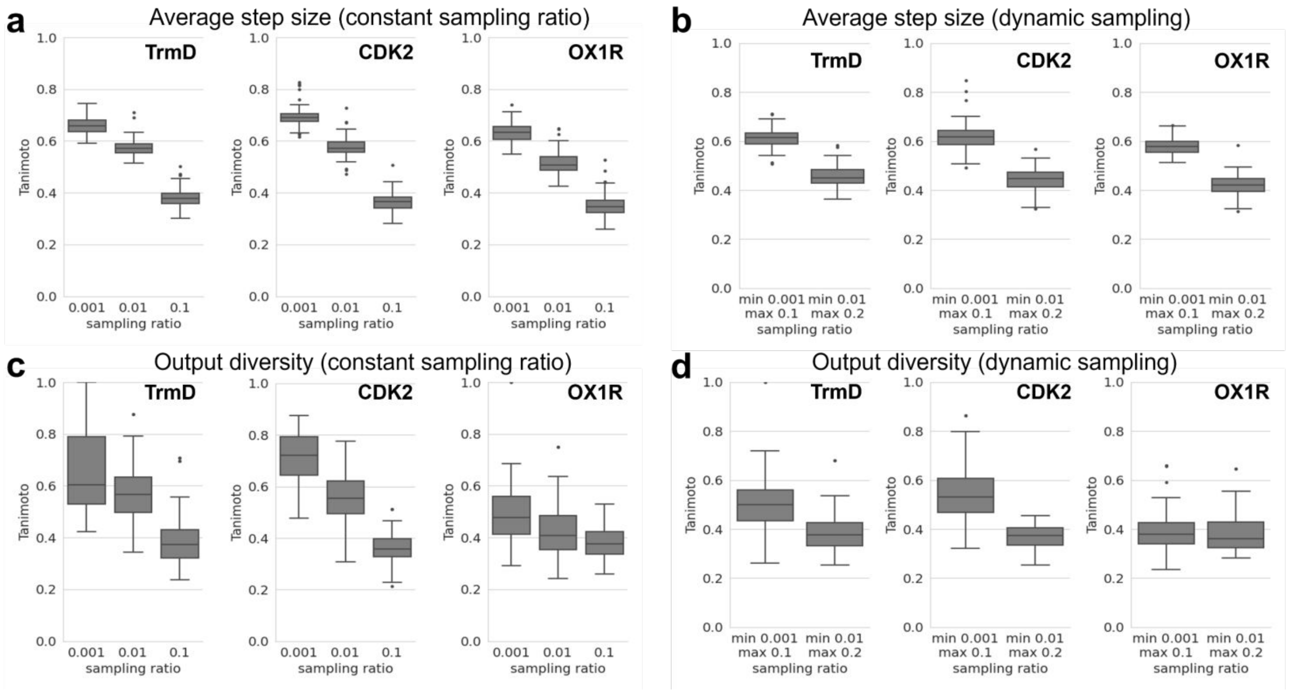

Figure S2. Impact of sampling ratio on step size and output diversity across different protein targets. The step size was defined as the Tanimoto similarity between consecutive accepted ligand candidates, with the average taken for 100 independent design routes. (a) Average step size distributions for TrmD, CDK2, and OX1R at constant sampling ratios of 0.001, 0.01, and 0.1. A smaller sampling ratio results in higher Tanimoto similarity (smaller step size) between consecutive molecules, indicating more focused exploration. (b) Average step size distributions at two sets of dynamic sampling ratios. Parameter set 1: min = 0.001, max = 0.1, step = 0.0005. Parameter set 2: min = 0.01, max = 0.2, step = 0.002. (c) Output diversity distributions for TrmD, CDK2, and OX1R at constant sampling ratios of 0.001, 0.01, and 0.1. Higher sampling ratios lead to more diverse outputs (lower maximum Tanimoto similarity between outputs). (d) Output diversity distributions at dynamic sampling ratios with the same thresholds as in panel b. In all cases, Tanimoto similarity is used to measure step size and diversity, with lower similarity indicating larger step size and greater diversity in molecular exploration.

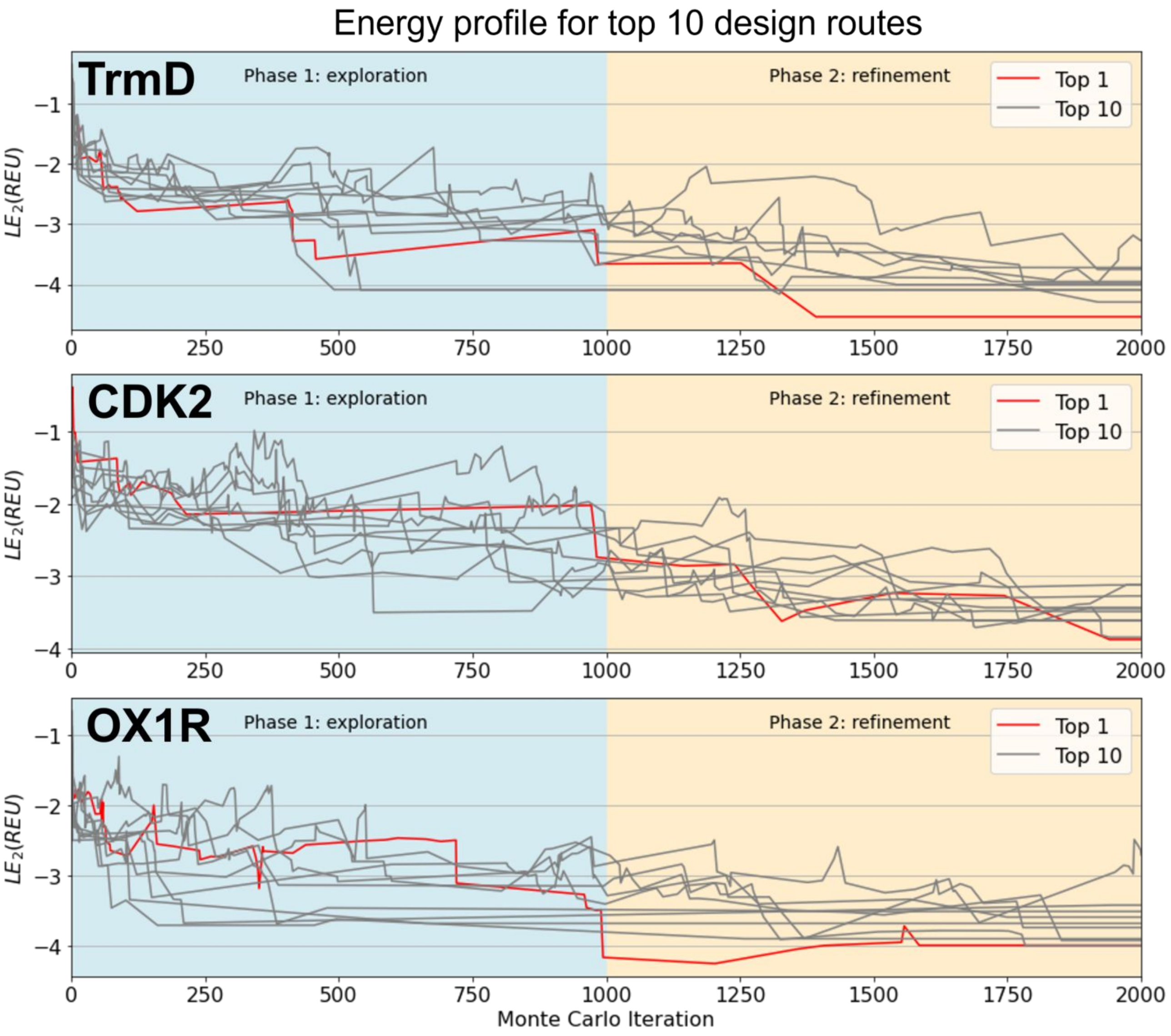

Figure S3. Energy profiles of the top 10 design routes across two optimization phases—exploration and refinement—for different protein targets. The best-scoring route is highlighted in red. In phase 1 (exploration), energy values fluctuate as diverse subspaces are sampled, with occasional uphill movements to escape local minima. In phase 2 (refinement), energy values stabilize as dynamic sampling is turned off, allowing the optimization process to converge towards energy minima.

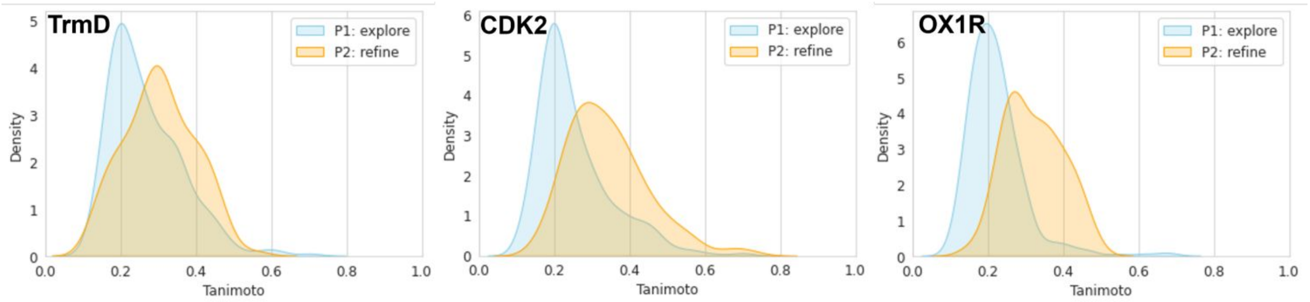

Figure S4. Tanimoto similarity distributions of the top 10 design routes across two optimization phases—exploration and refinement—for different protein targets. Similarity scores were calculated relative to the first accepted molecule sampled in each phase. In all targets, the exploration phase (blue) exhibits broader distributions and lower similarity scores, reflecting greater chemical diversity. The refinement phase (orange) shows narrower distributions and higher similarity scores, indicating focused optimization toward structurally related low-energy solutions.

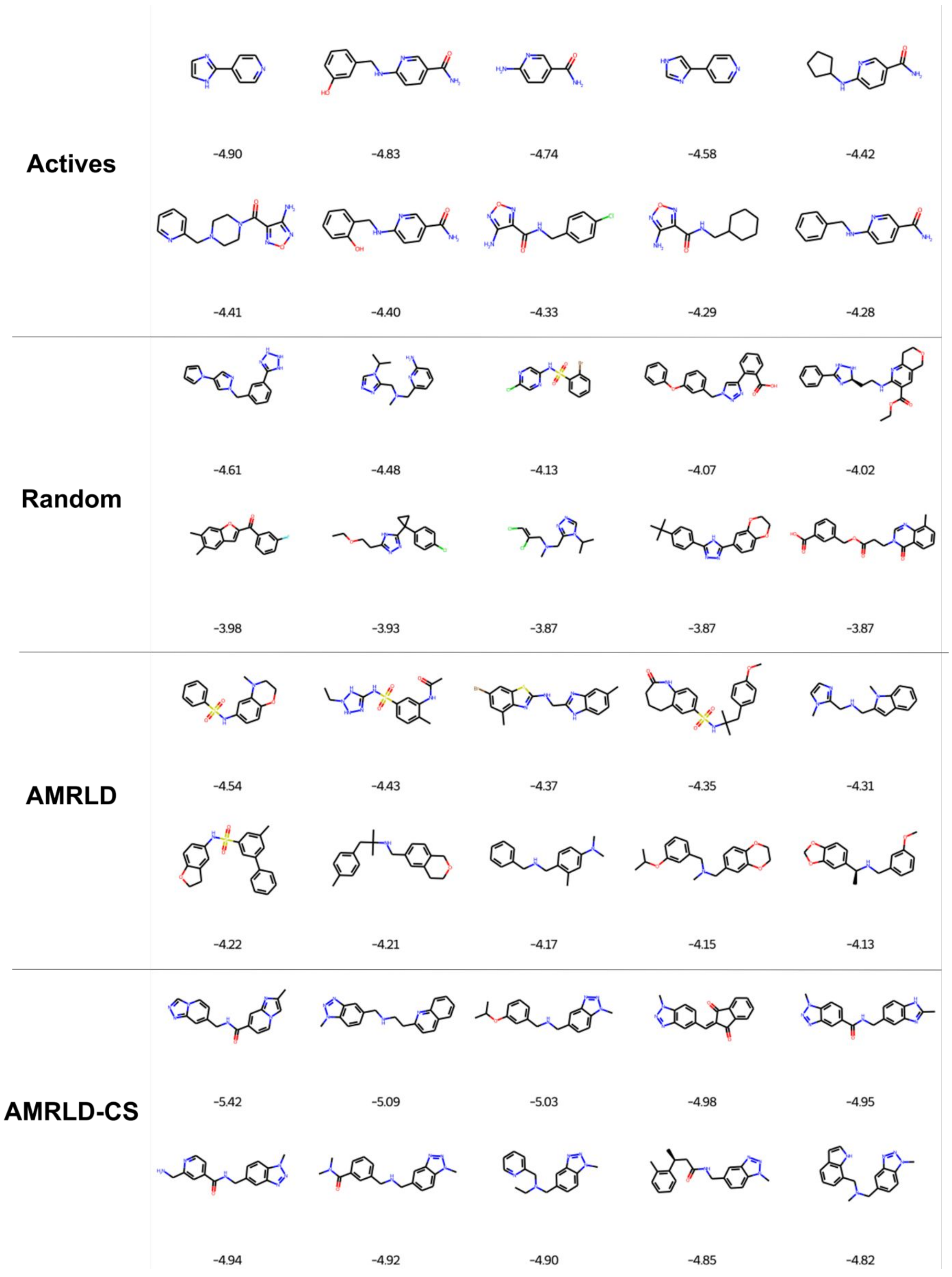

Figure S5. Top-scoring molecules by LE_2(REU) for TrmD.

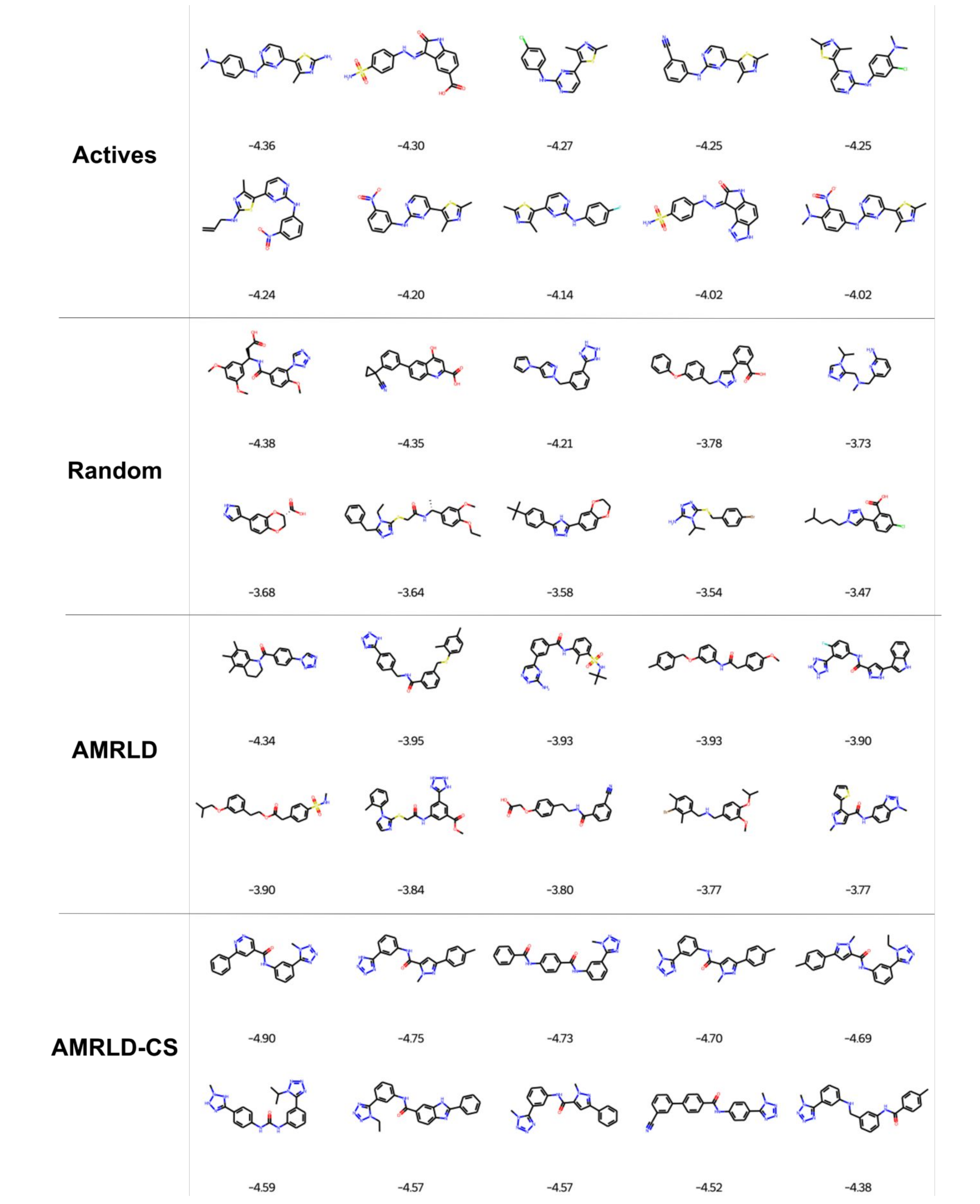

Figure S6. Top-scoring molecules by LE_2(REU) for CDK2.

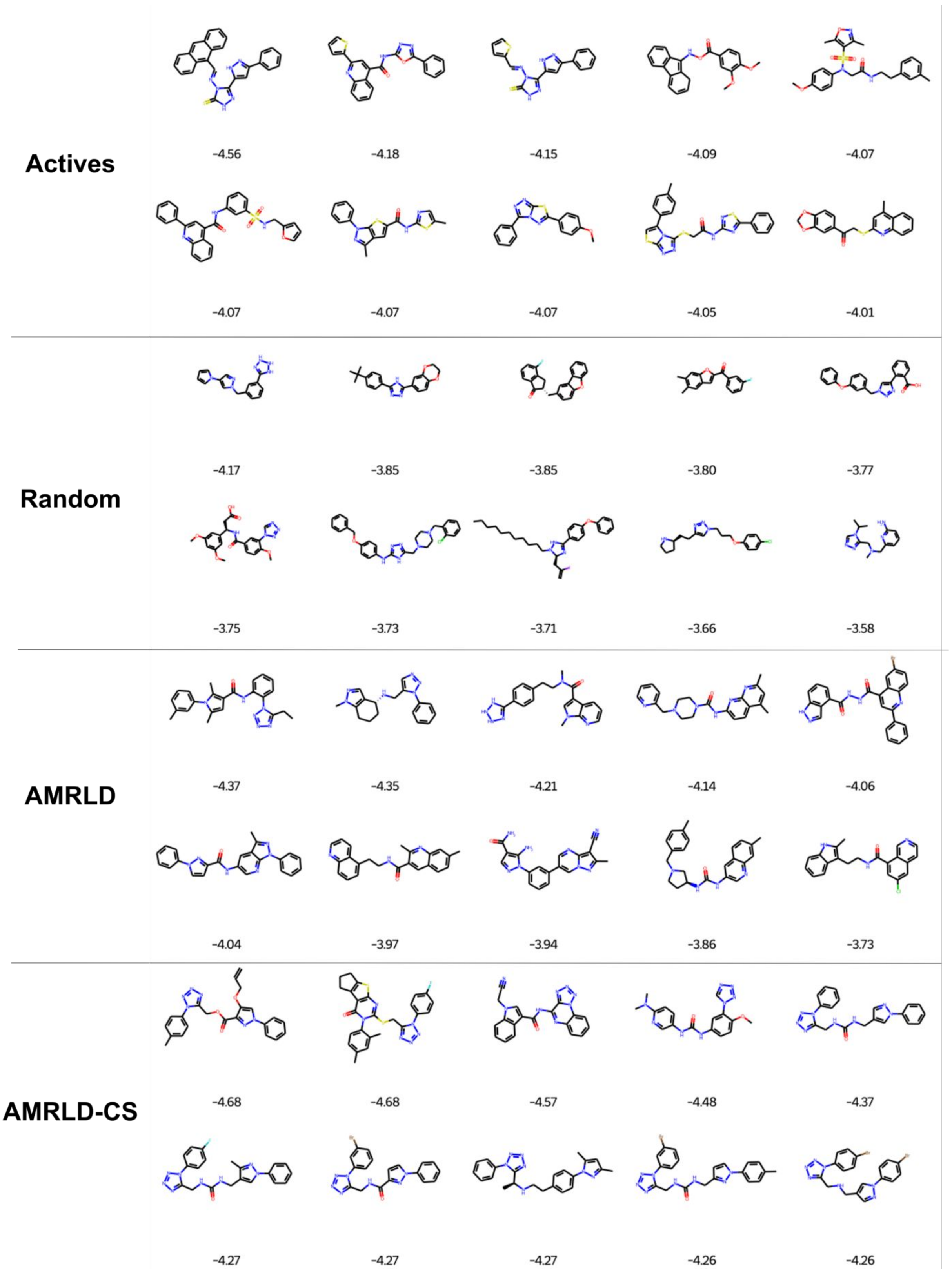

Figure S7. Top-scoring molecules by LE_2(REU) for OX1R.

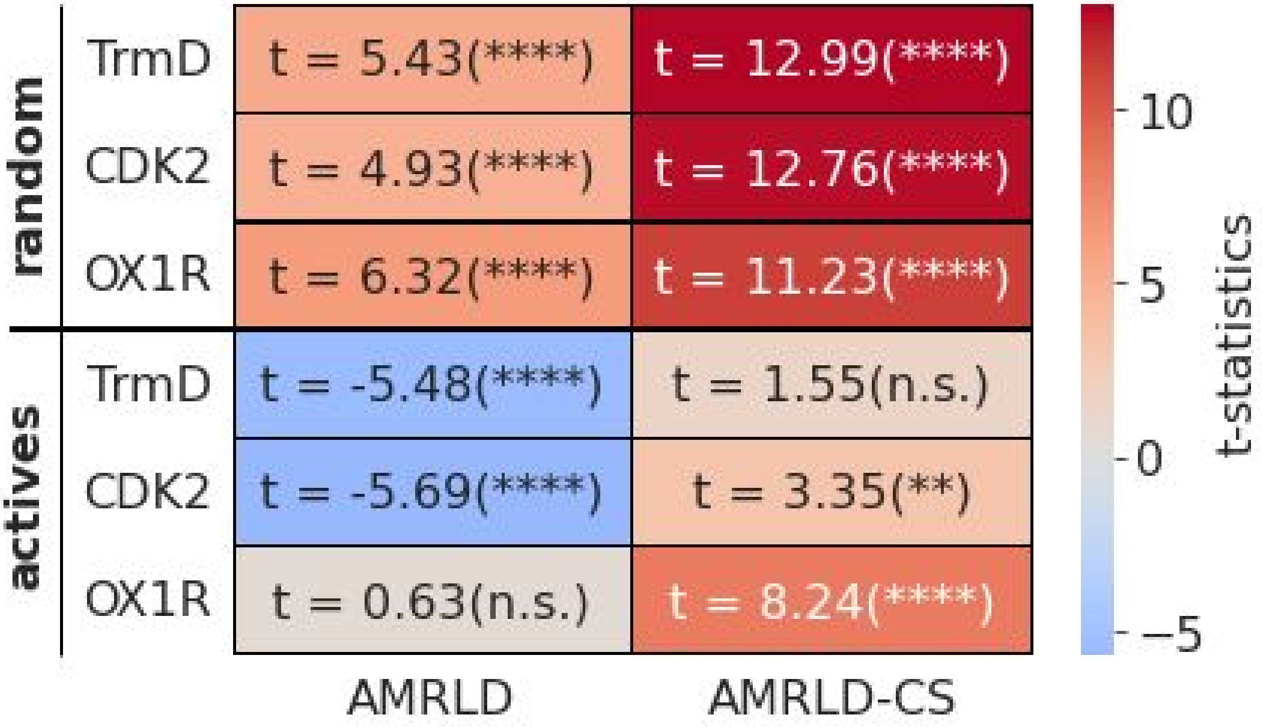

Figure S8. Statistical comparison of RosettaLigand docking scores between designed molecules and control groups across three protein targets. The heatmap shows t-statistics from two-sided Student t-test comparing the LE_2(REU) of AMRLD and AMRLD-CS designed molecules against random baseline and known actives. Positive t-values indicate that the designed molecules are better compared to the control group, while negative t-values indicate the opposite. Each cell is annotated with the corresponding t-statistic and significance level (n.s.: $p \geq 0.05$, not significant; *: $p < 0.05$; **: $p < 0.01$; ***: $p < 0.001$; ****: $p < 0.0001$). Color intensity reflects the magnitude and direction of the t-statistics.

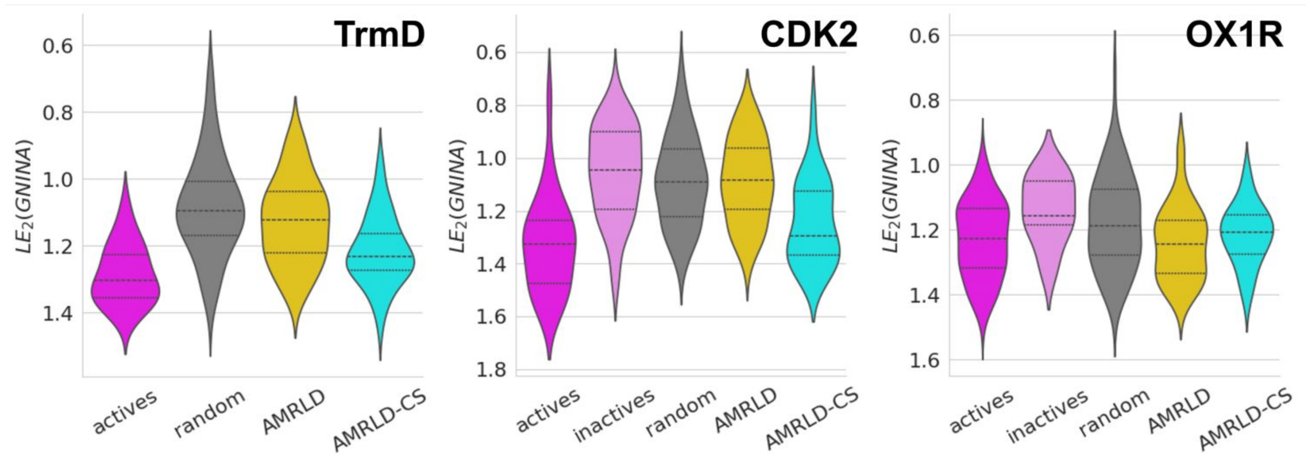

Figure S9. The GNINA CNNaffinity distribution for TrmD, CDK2, and OX1R. The affinity scores were normalized by the number of heavy atoms to account for molecular size. The y-axis is inverted to maintain consistency with Figure 3, where scores more towards the bottom indicates better molecules.

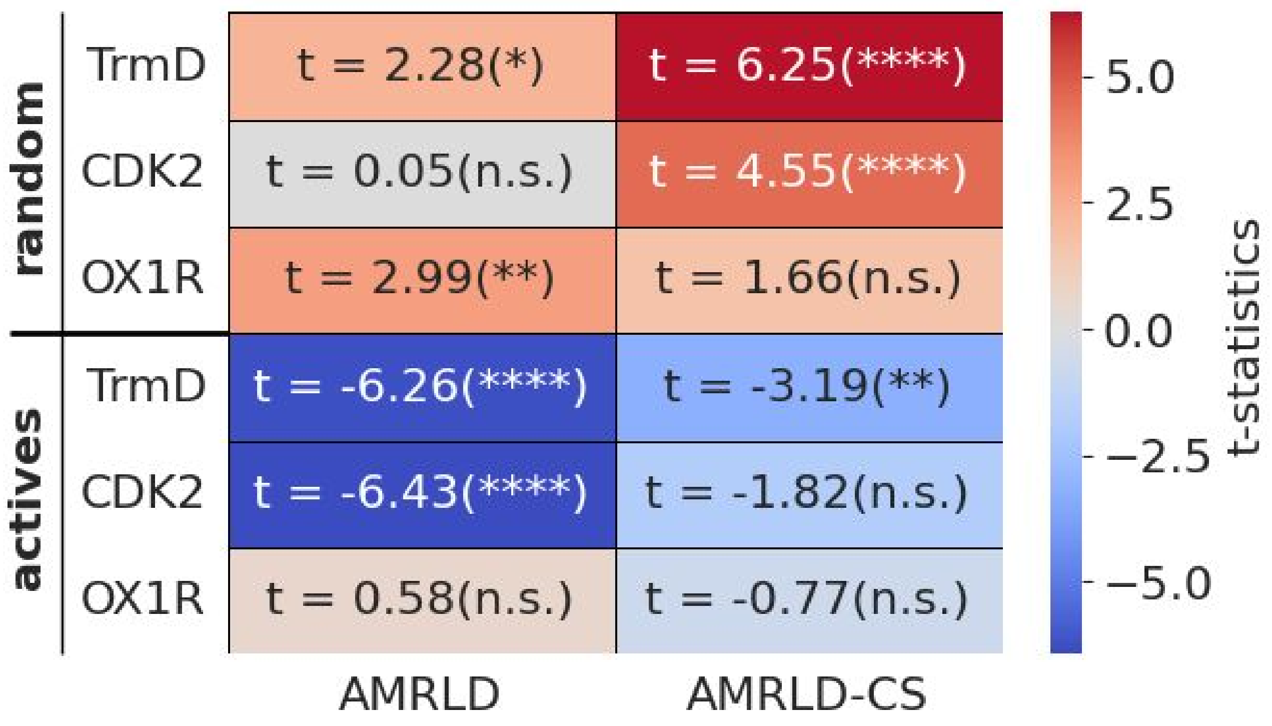

Figure S10. Statistical comparison of GNINA CNNaffinity between designed molecules and control groups across three protein targets. The heatmap shows t-statistics from two-sided Student t-test comparing the LE_2(GNINA) of AMRLD and AMRLD-CS designed molecules against random baseline and known actives. Positive t-values indicate that the designed molecules are better compared to the control group, while negative t-values indicate the opposite. Each cell is annotated with the corresponding t-statistic and significance level (n.s.: $p \geq 0.05$, not significant; *: $p < 0.05$; **: $p < 0.01$; ***: $p < 0.001$; ****: $p < 0.0001$). Color intensity reflects the magnitude and direction of the t-statistics.

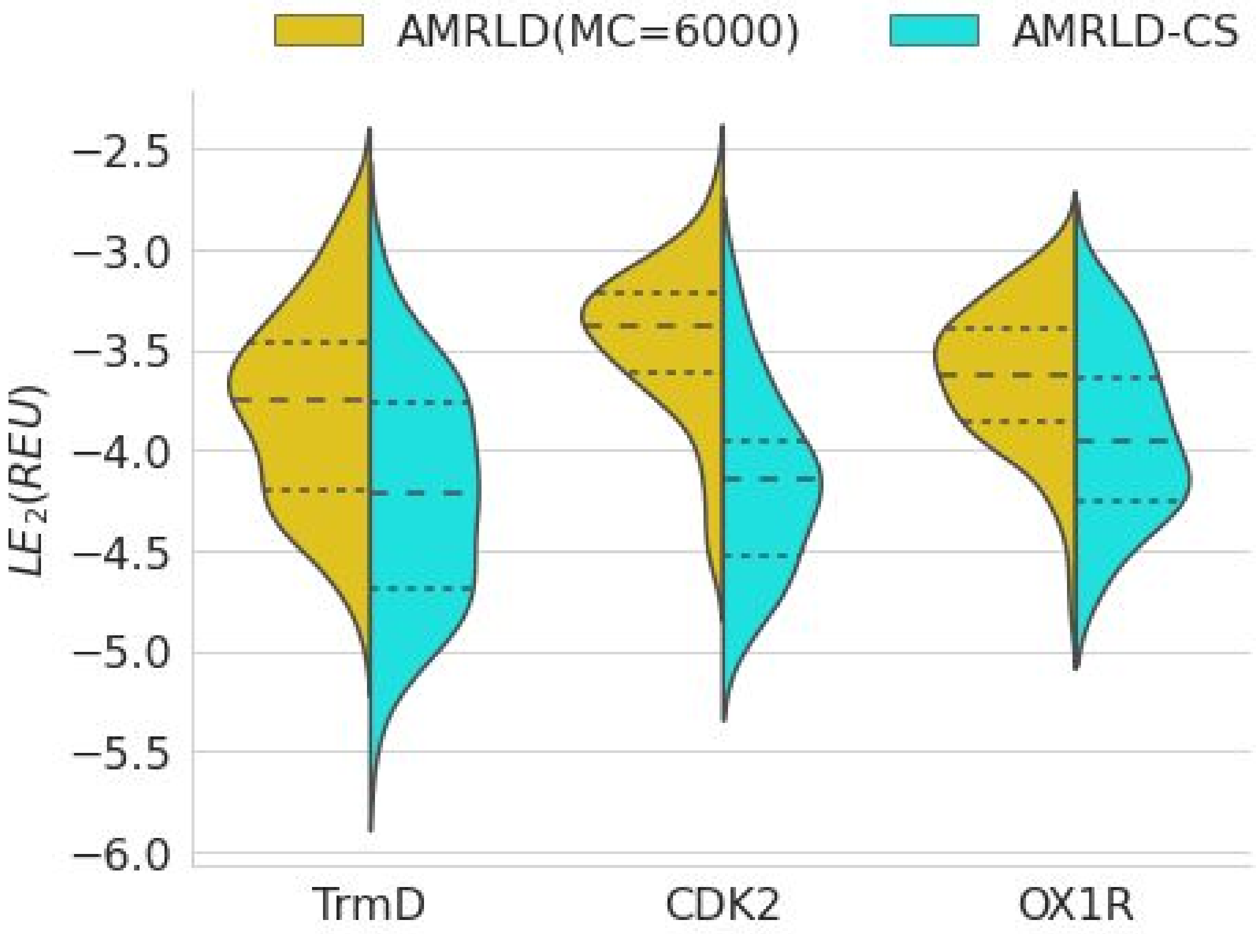

Figure S11. Comparison of cascaded sampling (AMRLD-CS) and extended single-round AMRLD (MC=6000). Violin plots show the distribution of LE_2(REU) scores for the designed ligands across three targets (TrmD, CDK2, OX1R). The AMRLD-CS workflow (cyan) consists of three rounds of standard RosettaAMRLD optimization, each set to 2000 Monte Carlo iterations (1000 exploration/1000 refinement), where top-performing ligands are selected and propagated to the next round. In contrast, the extended single-round AMRLD (yellow) is set to 6000 Monte Carlo iterations, with half/half split in exploration and refinement phases (3000 exploration/3000 refinement).

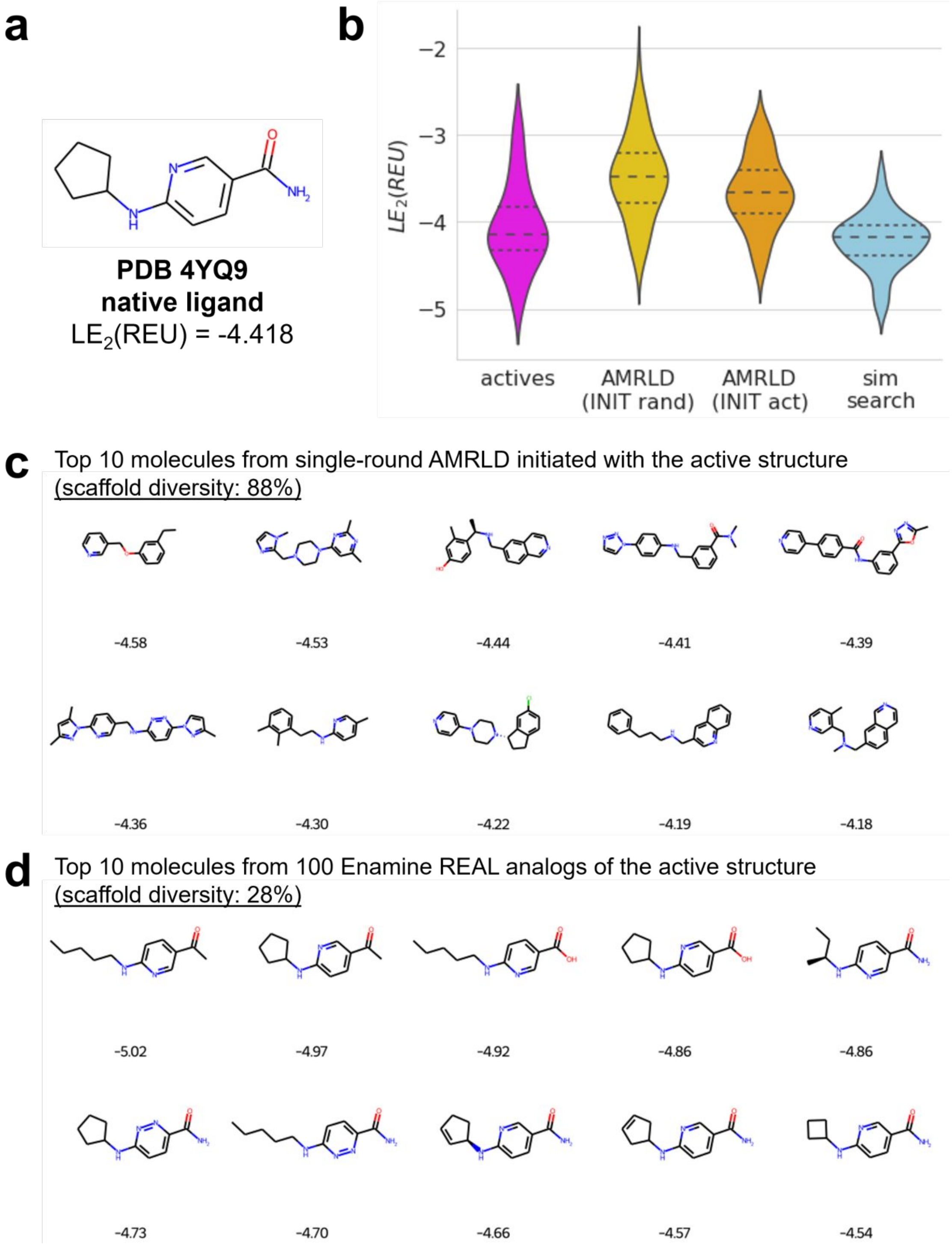

Figure S12. (a) The active structure used both as reference for similarity search and initial molecule for AMRLD lead optimization. (b) Comparison of docking scores between known actives, AMRLD outputs initiated with a poorly scoring random compound, AMRLD outputs initiated with an active, and similarity search of active analogs. The top 10 molecules from AMRLD lead optimization and similarity search, labelled with docking scores, are shown in (c) and (d) respectively, with scaffold diversity indicated for each set.

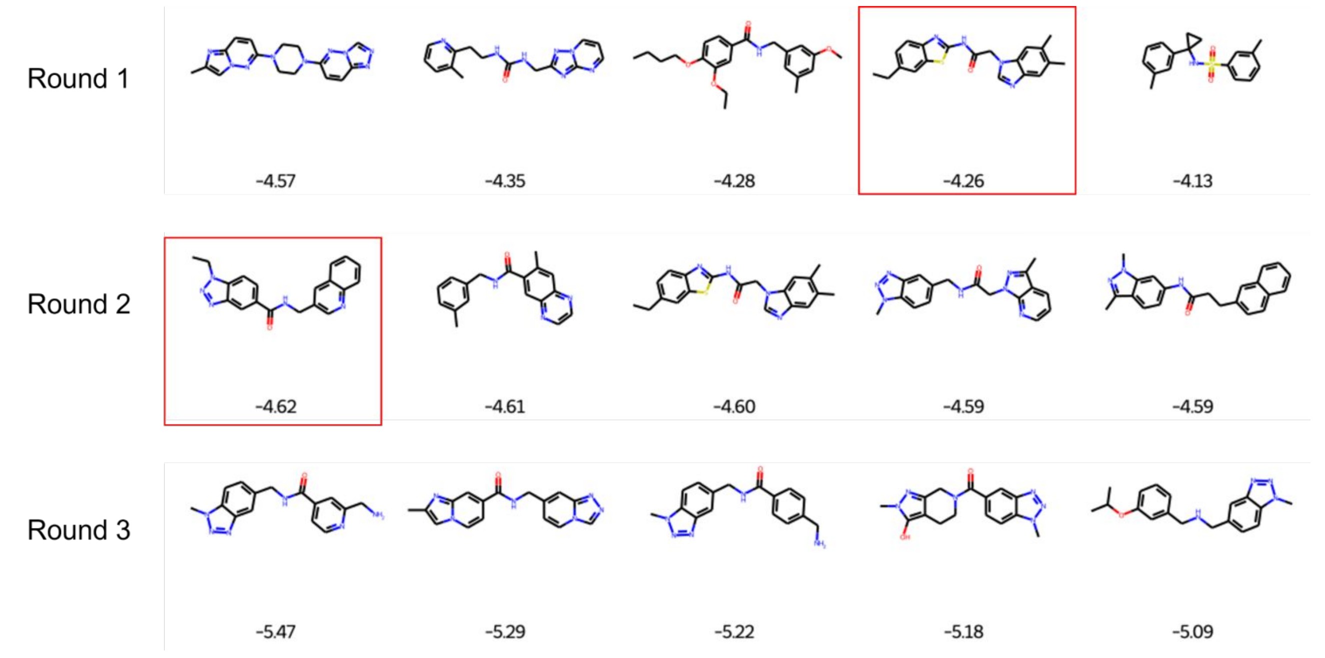

Figure S13. Top 5 designs by LE_2(REU) from each round of the cascaded sampling workflow for TrmD. The chosen starting entities for the next round are highlighted in red.

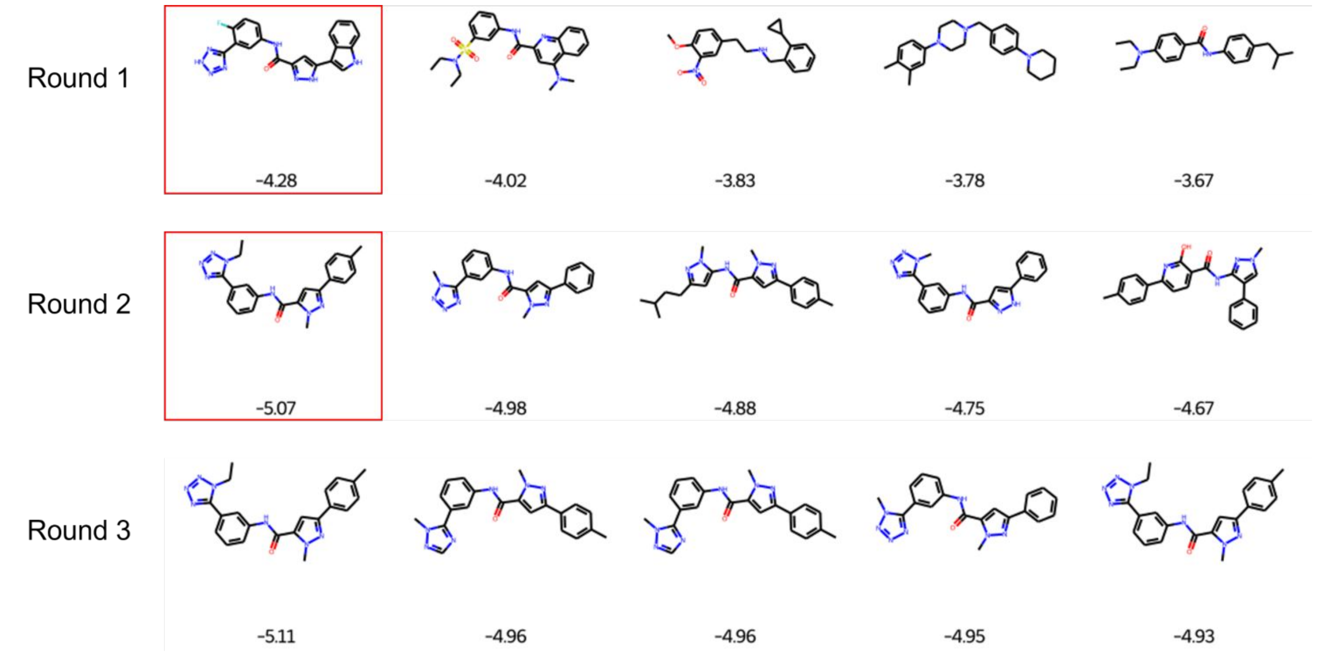

Figure S14. Top 5 designs by LE_2(REU) from each round of the cascaded sampling workflow for CDK2. The chosen starting entities for the next round are highlighted in red.

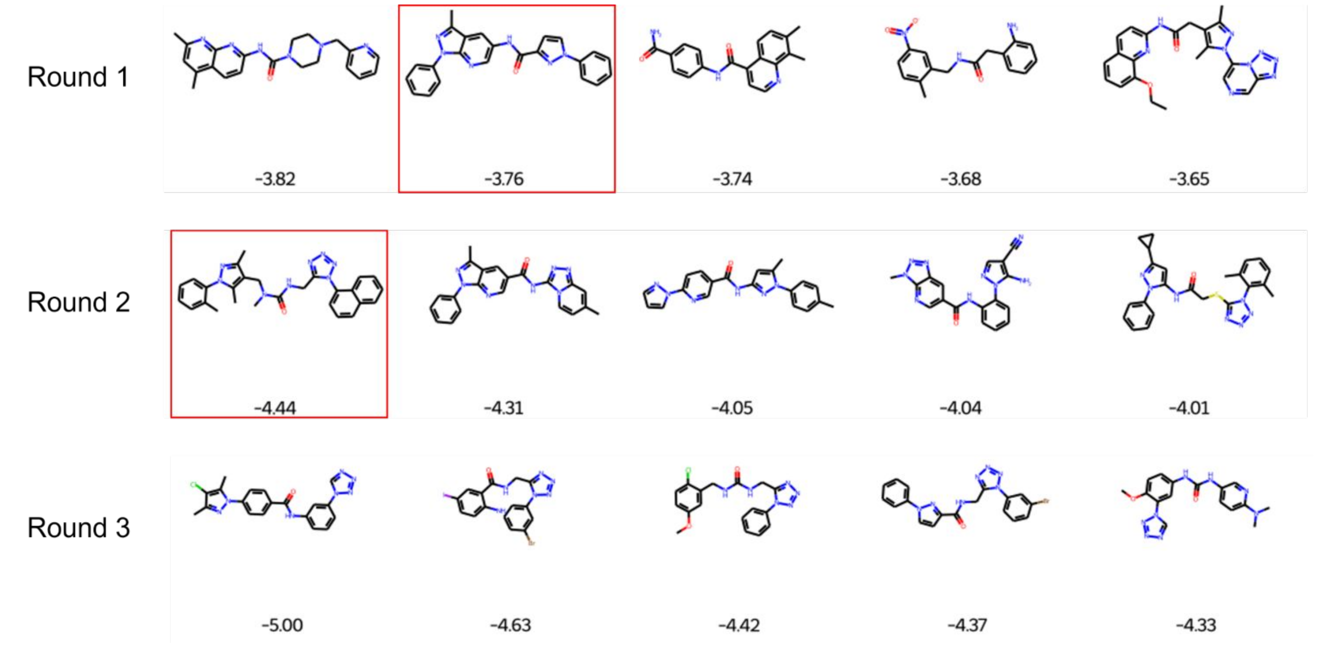

Figure S15. Top 5 designs by LE_2(REU) from each round of the cascaded sampling workflow for OX1R. The chosen starting entities for the next round are highlighted in red.
