# Supplementary material for: RosettaAMRLD: A Reaction-Driven Approach for Structure-Based Drug Design from Combinatorial Libraries with Monte Carlo Metropolis Algorithms
Source: J Chem Inf Model. 2025 Jun 11;65(12):5945–59. doi: 10.1021/acs.jcim.5c00497 (PMC12199295; doi:10.1021/acs.jcim.5c00497)
Supplement: Supplementary file 2 [file ci5c00497_si_002.zip › RosettaAMRLD_protocol_capture/RosettaAMRLD_Protocol_Capture.pdf]

RosettaAMRLD Protocol Capture

The following provides an example for using the Rosetta Automated Monte Carlo Reaction-based Ligand Design (RosettaAMRLD) protocol. Rosetta can be obtained through www.rosettacommons.org.

This protocol capture together with all input files and example outputs can be found in the `demos/protocol_capture/RosettaAMRLD/` directory in Rosetta.

File organization

This protocol capture and the provided scripts follows the directory structure below:

```
<Workspace>
  <inputs>
  <production>
  <scripts>
  <slurm>       # (optional) for running on cluster
</Workspace>
```

Protein and ligand files

The required structural files are as follows:

1. **A protein-ligand complex in PDB format.** This is the target structure with the starting ligand inside the pocket. It is highly recommended to dock the starting ligand first before running this protocol. The previously documented RosettaLigand docking protocol can be performed to get a reliable starting complex for this protocol.
2. **The starting ligand in SDF format**. The name of this ligand in the first line of the SDF file should match the ligand's three-letter code in the complex PDB file.
3. **The target protein in PDB format.** This is only required for the cascaded sampling workflow. This can be the input target structure for the RosettaLigand docking protocol, or can be the after-docking protein structure extracted from the above input complex. Other non-Rosetta structures are highly recommended to follow the relax protocol before running RosettaAMRLD.

The target structure used in this example is cyclin-dependent kinase 2 (CDK2) with PDB ID 4EK4. The co-crystalized ligand was removed and the chain A of the protein model was obtained.

```
path/to/Rosetta/main/tools/protein_tools/scripts/clean_pdb.py 4EK4 A
```

A constrained relax protocol in Rosetta was applied (Nivón LG, Moretti R, Baker D (2013) A Pareto-Optimal Refinement Method for Protein Design Scaffolds. PLOS ONE 8(4): e59004. https://doi.org/10.1371/journal.pone.0059004). Check RosettaCommons documentation for a

full list of available options (https://docs.rosettacommons.org/docs/latest/application_documentation/structure_prediction/relax).

```
mkdir relax/
path/to/Rosetta/main/source/bin/relax.default.linuxgccrelease -s 4EK4_A.pdb \
    -out:pdb -out:path:all relax/ -nstruct 100 -default_max_cycles 200 \
    -relax:constrain_relax_to_start_coords -ramp_constraints false \
    -multiple_processes_writing_to_one_directory
```

The 100 relaxed models were sorted by `total_score` and the best relaxed structure was renamed as `CDK2.pdb`.

```
sort -nk2 relax/score.sc | head -n 1
cp relax/4EK4_A_0090.pdb CDK2.pdb
```

The starting ligand for design is a poorly-scoring random compound, named as `rand39.sdf`. If the user has a desired starting ligand in SMILES format or PDB format, OpenBabel can be used to convert the molecule to SDF format.

```
obabel -:"[C@H]1(NC(=O)N[C@H](CC)C(F)(F)F)CCCN(C1)S(=O)(=O)c1ccc(Br)cc1" \
    -O rand39.sdf --title "LIG"
obabel rand39.pdb -O rand39.sdf --title "LIG"
```

This ligand was docked into the relaxed target structure following the RosettaLigand standard protocol (Lemmon, G., & Meiler, J. (2012). Rosetta ligand docking with flexible XML protocols. *Methods in Molecular Biology*, *819*, 143–155. https://doi.org/10.1007/978-1-61779-465-0_10). The best-scoring docked model by interface score (`interface_delta_X`) was renamed as `CDK2_rand39.pdb`. These files can be found in `/inputs/`.

Reaction and reagent files

In RosettaAMRLD, the reaction file defines the chemical reactions that can occur during ligand design and the reagent file provides the building blocks (reagents) that will participate in these reactions. RosettaAMRLD reads in reagents as Simplified Molecular Input Line Entry System (SMILES) and reactions as SMILES arbitrary target specification (SMARTS). Each reaction is identified by a unique reaction ID, the number of components (reactants), and the SMARTS notation of the reaction. Each reagent entry is identified by the SMILES notation, a unique reactant ID, the n-th component of the reaction, and the corresponding reaction ID.

The reported results used the Enamine REAL space (2023-01 version) which contains more than 36 billion compounds. However, due to the non-disclosure agreement, we cannot distribute the particular library for this protocol capture. The user may consider accessing the Enamine library through BioSolveIT (https://www.biosolveit.de/infiniSee_xREAL).

Here, we composed a small library from ChEMBL (https://www.ebi.ac.uk/chembl/) for proof of concept. We downloaded all synton-size (100-300 Da) and neutral small molecules. From this set of

molecules, we selected 74.5k molecules in total as reagents that can undergo one of four reactions: amide formation, arylation, ether formation, and reduction amination. The reaction and reagent files for this small library can be found under `/Workspace/` as `reactions.txt` and `reagents.txt`.

RosettaScripts XML file

The XML file describes the specific protocol to be performed by Rosetta and allows the user to customize its components. We provide the settings for the reported results in `drug_design.xml` under `/Workspace/`. A large part of the XML tags are based on the RosettaLigand standard docking protocol and the introduction to these tags can be found in its published article (Lemmon, G., & Meiler, J. (2012). Rosetta ligand docking with flexible XML protocols. *Methods in Molecular Biology*, *819*, 143–155. https://doi.org/10.1007/978-1-61779-465-0_10). We used the RosettaLigand scoring function to generate the reported results, but the user may substitute with any Rosetta scoring function.

There are some new XML tags specific to this protocol:

1. **ReactionBasedAnalogSampler**
   This is the proposal generation step where a reaction product is sampled from a given chemical library by similarity to a reference molecule (for the first Monte Carlo iteration, this is the input ligand; for later iterations, the reference is the prior accepted molecule). The path to the chemical library (relative to this XML script) is required.

   The sampling ratio, a value between 0 and 1, controls how the sampling weights are distributed along the similarity rank (the top *x* proportion of all molecules accounts for *1-x* of the total weight). A low sampling ratio concentrates the weights on top-ranking entities, favoring the selection of more similar molecules, while a higher sampling ratio places more weight on lower-ranking entities, promoting the selection of less similar molecules. When incorporated into Monte Carlo iterations, the sampling ratio directly influences the step size and scope of exploration in the chemical space. A higher sampling ratio allows broader exploration, while a lower ratio focuses on local refinement. Typically, it is adjusted by orders of magnitude (e.g., 0.1, 0.01, 0.001) during parameter tuning. However, we recommend selecting values no higher than 0.25 to maintain the theoretical validity within a two-decimal place approximation. The `sampling_ratio` defines a constant ratio unless dynamic sampling is enabled, in which case it defines the ratio only for the first Monte Carlo iteration.

   Dynamic sampling provides an adaptive scheme for the sampling ratio and is utilized to search more efficiently in a wide scope. This approach is particularly useful when no known binders are available or when the design goal involves scaffold hopping from a reference structure. The user defines a range and a rate of increase for the sampling ratio. At accepted Monte Carlo iterations, the sampling ratio resets to its defined `min` value and at rejected iterations, the ratio increases by the specified `step`. If consecutive rejections cause the ratio to reach the defined `max`, it will remain capped at `max` until the next accepted iteration. Dynamic sampling can be disabled in later Monte Carlo iterations to help resting the exploration to a local minimum. By setting `OFF_after_n_step`, the sampling ratio reset to the `base` value after the specified number of iterations, remaining constant to the end of the search.

In the second sampling stage, where the final product is sampled from a set of candidates, a minimum number of candidates must be generated before selection. This minimum threshold can be adjusted using the `minCandidates` parameter. Setting a higher threshold reduces the likelihood of duplicate scaffolds appearing in the same molecule, which can occur when the library contains an uneven distribution of fragments favoring one scaffold over others. However, a higher threshold will also increase the sampling time. In practice, we recommend setting the threshold between 20 and 100.

```
<ReactionBasedAnalogSampler name=(string) reactions=(string) reagents=(string)
   sampling_ratio=(float) minCandidates=(int) >
   <DynamicSampling min=(float) max=(float) step=(float) OFF_after_n_step=(int)
      base=(float) />
</ReactionBasedAnalogSampler>
```

2. **LigandLocationFilter**
   This filter checks if the ligand's centroid is within a specified distance from the center of the pocket. A set of xyz coordinates is required for the particular input biological target. For this example, the center is set to the coordinates of atom CAL in the co-crystalized ligand 1CK of CDK2 structure 4EK4.

```
<LigandLocationFilter name=(string) chain=(char) radius=(float)>
   <center x=(float) y=(float) z=(float) />
</LigandLocationFilter>
```

3. **RDKitMetric**
   This is a filter that uses RDKit (https://www.rdkit.org/docs/GettingStartedInPython.html#list-of-available-descriptors) to calculate the specified metric for the ligand. The lower and upper thresholds are required for the filter to remove unwanted ligands.

```
<RDKitMetric name=(string) metric=(string) lower_threshold=(float)
   upper_threshold=(float) residue=(string) />
```

4. **DrugDesignMover**
   This is the Monte Carlo Metropolis framework that performs iterations of chemistry operations to generate new ligands and score them inside the pocket. The framework requires a redocker and a scorer to emplace the new ligands and calculate an interface score. While the user may use any suitable setting as for a docking protocol, in our reported results, we set the redocker to be a single-cycle high-resolution docking followed by a final minimizer, a one-shot docker. As for the scorer, the interface energy is normalized by the number of heavy atoms in the ligand and paired with a cLogP bandpass filter to penalize undesired nonpolar molecules. Examples and details of these scoring settings can be found in the filters section of the XML script. The energy for the metropolis criterion by default considers ligand efficiency. If the user wish to turn this off, besides setting

`lig_efficy` to `false`, the scorer also needs the normalization part removed. In the provided XML script, set `equation="sig*E"` to turn off ligand efficiency consideration. Similarly, the cLogP bandpass filter can also be removed by setting `equation="E"`, in which case the raw interface energy is used for the metropolis criterion.

```
<CalculatorFilter name="ligE" equation="sig*E/sqrt" threshold="0" >
    <Var name="sqrt" filter="nHeavy_sqrt" />
    <Var name="E" filter="interface" />
    <Var name="sig" filter="CLP_Sigmoid" />
</CalculatorFilter>
```

The DrugDesignMover allows inclusion of pre-filters and post-filters to remove certain ligands before and after re-docking. The RDKitMetrics introduced above, for example, can be used here to reject ligands with unwanted physicochemical properties. The LigandLocationFilter, on the other hand, is a post-filter to reject any ligand that is redocked outside the pocket.

The number of Monte Carlo iterations is set by `trials` and increasing this number proportionally increases computational time. The temperature parameter in the Metropolis criterion controls the acceptance rate. This is set according to the scale of Rosetta energy function normalized by the number of heavy atoms. Unless the user employs a scoring function of different scale or turns off the ligand efficiency option, the temperature parameter in the provided XML should be the optimal setting for most cases (if ligand efficiency is turned off, consider increasing temperature to 0.5).

The chemistry operations performed in each iteration have three types: major, before, and after. A major chemistry operation modifies the ligand into a new ligand, and a list of such operations may exist, where a random operation is selected by the assigned weights every iteration. For the current RosettaAMRLD, `ReactionBasedAnalogSampler` is the only major chemistry operation. The "before" and "after" chemistry operations are always performed before and after the major operation, respectively. For example, a rotamer generation step can be an "after" chemistry operation to generate conformations for the new ligand.

```
<DrugDesignMover name=(string) chain=(char) scorer=(string) redocker=(string)
    prefilter=(string) postfilter=(string) trials=(int) temperature=(float) lig_efficy=[true|false]>
    <Add chemistry=(string) weight=(float) />
    <Before chemistry=(string) />
    <After chemistry=(string) />
</DrugDesignMover>
```

Rosetta options file

The provided options file `drug_design_options.txt` under `/Workspace/` contains some standard docking options. A full available options list can be found on the Rosetta documentation website.

Running RosettaAMRLD

1. Create an output folder under `/production/`.

```
mkdir -p production/rand39
```

2. Run RosettaAMRLD under `/Workspace/`. Here as an example, we collect 1 design only (`nstruct 1`). In practice, the protocol is usually run on a cluster so that tens or hundreds of designs can run in parallel. Since the process is stochastic, this single design the user get in this example may or may not be a decent one, but the user should be able to observe some optimization in energy in the result files.

```
path/to/Rosetta/main/source/bin/rosetta_scripts.linuxgccrelease @drug_design_options.txt \
    -parser:protocol drug_design.xml \
    -s inputs/CDK2_rand39.pdb \
    -extra_res_mol inputs/rand39.sdf \
    -out:path:all production/rand39/ \
    -nstruct 1
```

3. Once the run finishes, there should be three files in the output folder: 1) `CDK2_rand39_0001.pdb` — the design pose; 2) `score.sc` — the Rosetta score file containing the detailed breakdown energies of the design pose; 3) `CDK2_rand39_0001.log` — the log file which records all accepted ligands during the design (organized in this format below). The last lines of the log file are the reagent SMILES that formed the output product.

```
<iteration>,<raw interface energy>,<normalized interface energy>,<SMILES>
...
<MC statistics>
The best scoring ligand is <SMILES>
Fragment0: <SMILES>
Fragment1: <SMILES>
```

Running on a cluster

An example SLURM script `run.slurm` for running on a cluster can be found under `/Workspace/`. Replace the `<protein>` and `<ligand>` with the corresponding protein and ligand filenames (excluding file extensions). The script is set to run 100 designs in parallel by default. To adjust the number of designs, modify the job array parameter (`--array=1-[x]`) to the desired count. Each output file will be prefixed with the corresponding job array number. If the user increases `-nstruct` to generate more designs per job, the runtime must be adjusted proportionally.

Analysis

The provided scripts for post-processing and analysis require the following packages: RDKit, OpenBabel, Pandas, and Matplotlib. Here we assume the user has these packages installed.

Navigate to the `/production/` folder. Generate a summarized and ranked score file for the designs.

```
cd production/
../scripts/post_design.sh rand39
```

The script outputs two files: a summarized score file and a PNG file of the top designs.

- The summarized score file `design_scores.sc` is saved in the corresponding ligand output directory (`rand39/`). This file contains the filename, total score, raw interface energy (`interface_delta_X`), normalized interface energy (`LE2(REU)`), and SMILES for all designs, sorted by normalized interface energy.
- The PNG file (`rand39.png`), output to the `/production/` directory, displays the 2D structures of up to the 10 best designs as well as their normalized interface energies. To visualize more ligands, run the following script again with the desired number.

  ```
  ../scripts/draw_top_design.py -i rand39/design_scores.sc --output rand39.png --topN [n]
  ```

Navigate to the ligand output folder and plot the energy profile for each design.

```
cd rand39/
../../scripts/plot_scores_over_time.py *.log --separate_image --mc 2000 --column 2
```

This script plots energy versus the accepted Monte Carlo iteration number and saves a PNG file for each design in the directory. An ideal energy profile shows a downward trend over time, with occasional hill-climbing. This visualization can help assess if further tuning of design parameters is needed.

By default, the script generates separate images for each design. To plot all designs in a single image, exclude the `--separate_image` option. The `--mc` option specifies the total number of iterations in the design, while the `--column` option indicates which score column to plot (defaulting to normalized interface energy; set to `1` to plot raw interface energy).

Cascaded Sampling Workflow

This workflow extends the promising design routes to explore a wider scope on those directions, discovering better molecules in distant subspaces. This is essentially running multiple rounds of RosettaAMRLD with top-scoring designs from each round chosen as new starting ligands in the next round of optimization.

After a round of RosettaAMRLD, visually inspect the top-scoring ligands and select a set of 1-5 ligands with diverse scaffolds for cascaded sampling in the next round. Run the following script under the `/production/` directory to prepare these ligands for the next round input. Make sure a cleaned protein PDB file is in the `/inputs/` directory.

```
cd production/
../scripts/cascaded_prepare.sh rand39 1 CDK2
```

The first command line option locates the current round output path (in this case named as the starting ligand). The second command line option specifies the number of top-scoring ligands to prepare (for example if the user's set consists of the 1st, 3rd, 5th ligands, set this option to `5` so that all five ligands are prepared). The third command line option refers to the target protein PDB filename.

This script prepares all the required structural input files for the next round of RosettaAMLRD in the `/inputs/` directory. Additionally, a PNG file with the 2D structures of the individual top ligands is also generated for record. To help keep track of various design routes and ligand series, an `Cascaded_README` file is generated under the `/production/` directory and is automatically updated every time the script is executed. See below for an example of this file.

```
=====Mon Oct 28 11:24:14 CDT 2024=====
Current ligand: rand39
Selected 1 ligands for cascaded sampling.
CDK2_rand39_0001 → rand39_LIG1
```

The top ligands are renamed as `<current_ligand>_LIGXX` where `XX` refers to its rank in score. As multiple rounds of optimization proceed, the ligand name also keeps track of the design route. Use the corresponding new ligand name to run the next round of RosettaAMRLD.

```
mkdir -p production/rand39_LIG1
path/to/Rosetta/main/source/bin/rosetta_scripts.linuxgccrelease @drug_design_options.txt \
    -parser:protocol drug_design.xml \
    -s inputs/CDK2_rand39_LIG1.pdb \
    -extra_res_mol inputs/rand39_LIG1.sdf \
    -out:path:all production/rand39_LIG1/ \
    -nstruct 1
```

In practice, this workflow can be repeated for 2-5 rounds or until no further improvement in energy is observed. In some cases the design routes may converge and structurally similar ligands are observed across different routes, indicating discovery of an optimal design within a relatively wide subspace.
