# Supplementary figures and images for: RosettaAMRLD: A Reaction-Driven Approach for Structure-Based Drug Design from Combinatorial Libraries with Monte Carlo Metropolis Algorithms
Source: J Chem Inf Model. 2025 Jun 11;65(12):5945–59. doi: 10.1021/acs.jcim.5c00497 (PMC12199295; doi:10.1021/acs.jcim.5c00497)

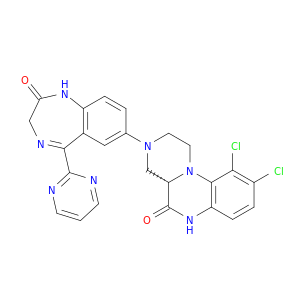

Supplement: Supplementary file 2 [file ci5c00497_si_002.zip › RosettaAMRLD_protocol_capture/inputs/rand39_LIG1.png]

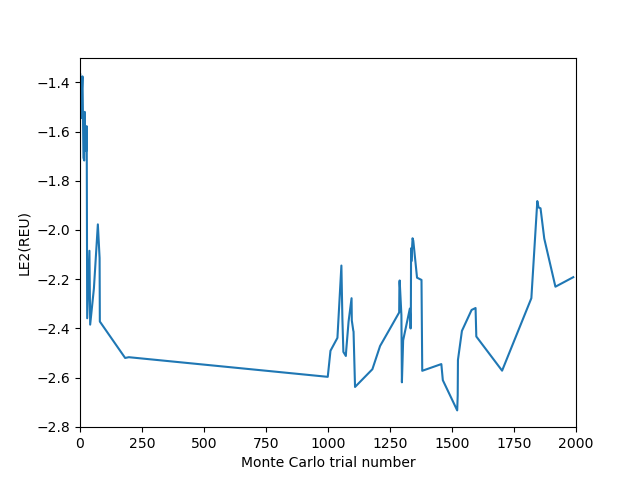

Supplement: Supplementary file 2 [file ci5c00497_si_002.zip › RosettaAMRLD_protocol_capture/production/rand39/CDK2_rand39_0001.png]

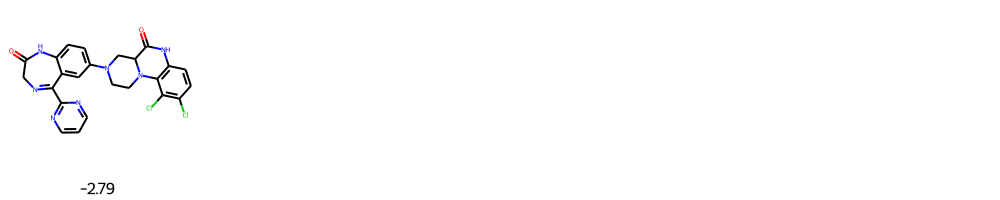

Supplement: Supplementary file 2 [file ci5c00497_si_002.zip › RosettaAMRLD_protocol_capture/production/rand39.png]

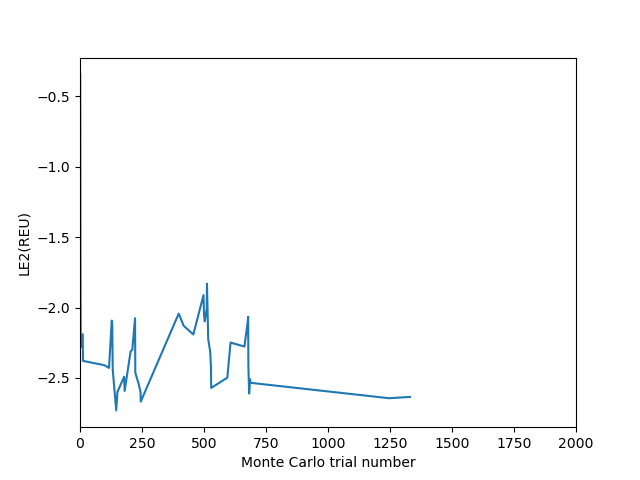

Supplement: Supplementary file 2 [file ci5c00497_si_002.zip › RosettaAMRLD_protocol_capture/production/rand39_LIG1/CDK2_rand39_LIG1_0001.png]

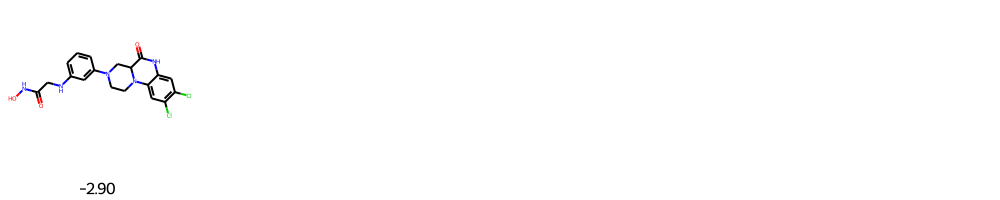

Supplement: Supplementary file 2 [file ci5c00497_si_002.zip › RosettaAMRLD_protocol_capture/production/rand39_LIG1.png]
